# Contrasting phylogeographic pattern among *Eudyptes* penguins around the Southern Ocean

**DOI:** 10.1038/s41598-018-35975-3

**Published:** 2018-11-30

**Authors:** M. J. Frugone, A. Lowther, D. Noll, B. Ramos, P. Pistorius, G. P. M. Dantas, M. V. Petry, F. Bonadonna, A. Steinfurth, A. Polanowski, A. Raya Rey, N. A. Lois, K. Pütz, P. Trathan, B. Wienecke, E. Poulin, J. A. Vianna

**Affiliations:** 1 Pontificia Universidad Católica de Chile, Facultad de Agronomía e Ingeniería Forestal, Departamento de Ecosistemas y Medio Ambiente, Vicuña Mackenna 4860, 7820436 RM Santiago, Chile; 2 Instituto de Ecología y Biodiversidad, Universidad de Chile, Departamento de Ciencias Ecológicas, Santiago, Chile; 3 Norwegian Polar Institute, Tromsø, N-9297 Norway; 4 DST/NRF Centre of Excellence at the Percy FitzPatrick Institute for African Ornithology, Department of Zoology, Nelson Mandela University, Port Elizabeth, 6031 South Africa; 5 Pontificia Universidade Católica de Minas Gerais, PPG in Vertebrate Zoology, Belo Horizonte, Brazil; 6 Universidade do Vale do Rio dos Sinos, Laboratório de Ornitologia e Animais Marinhos, Av. Unisinos 950, São Leopoldo, RS Brazil; 7 CEFE UMR 5175, CNRS, Université de Montpellier, Université Paul-Valéry Montpellier, EPHE, 1919 route de Mende, 34293 Montpellier cedex 5, Montpellier, France; 8 FitzPatrick Institute of African Ornithology, DST/NRF Centre of Excellence, University of Cape Town, 7700 Rondebosch, South Africa; 9 Present Address: RSPB Centre for Conservation Science, Royal Society for the Protection of Birds, David Attenborough Building, Pembroke Street, Cambridge, Cambridgeshire CB2 3QZ United Kingdom; 10 Australian Antarctic Division, 203 Channel Highway Kingston, Tasmania, 7050 Australia; 11 Centro Austral de Investigaciones Científicas – Consejo Nacional de Investigaciones Científicas y Técnicas (CADIC-CONICET), Bernardo Houssay 200, Ushuaia, Tierra del Fuego Argentina; 12 Instituto de Ciencias Polares, Ambiente y Recursos Naturales, Universidad Nacional de Tierra del Fuego, Yrigoyen 879, Ushuaia, Argentina; 13 Laboratorio de Ecología y Comportamiento Animal, Instituto de Ecologia, Genética y Evolución de Buenos Aires – Consejo Nacional de Investigaciones Científicas y Técnicas (IEGEBA-CONICET), Universidad de Buenos Aires, Buenos Aires, Argentina; 14 Antarctic Research Trust, Zürich, Am Oste-Hamme-Kanal 10, 27432 Bremervörde, Germany; 15 British Antarctic Survey, High Cross, Madingley Road Cambridge, UK CB3 0ET United Kingdom

## Abstract

Since at least the middle-Miocene, the Antarctic Polar Front (APF) and the Subtropical Front (STF) appear to have been the main drivers of diversification of marine biota in the Southern Ocean. However, highly migratory marine birds and mammals challenge this paradigm and the importance of oceanographic barriers. *Eudyptes* penguins range from the Antarctic Peninsula to subantarctic islands and some of the southernmost subtropical islands. Because of recent diversification, the number of species remains uncertain. Here we analyze two mtDNA (HVRI, COI) and two nuclear (ODC, AK1) markers from 13 locations of five putative *Eudyptes* species: rockhopper (*E. filholi, E. chrysocome*, and *E. moseleyi*), macaroni (*E. chrysolophus*) and royal penguins (*E. schlegeli*). Our results show a strong phylogeographic structure among rockhopper penguins from South America, subantarctic and subtropical islands supporting the recognition of three separated species of rockhopper penguins. Although genetic divergence was neither observed among macaroni penguins from the Antarctic Peninsula and sub-Antarctic islands nor between macaroni and royal penguins, population genetic analyses revealed population genetic structure in both cases. We suggest that the APF and STF can act as barriers for these species. While the geographic distance between colonies might play a role, their impact/incidence on gene flow may vary between species and colonies.

## Introduction

**Figure 1:**
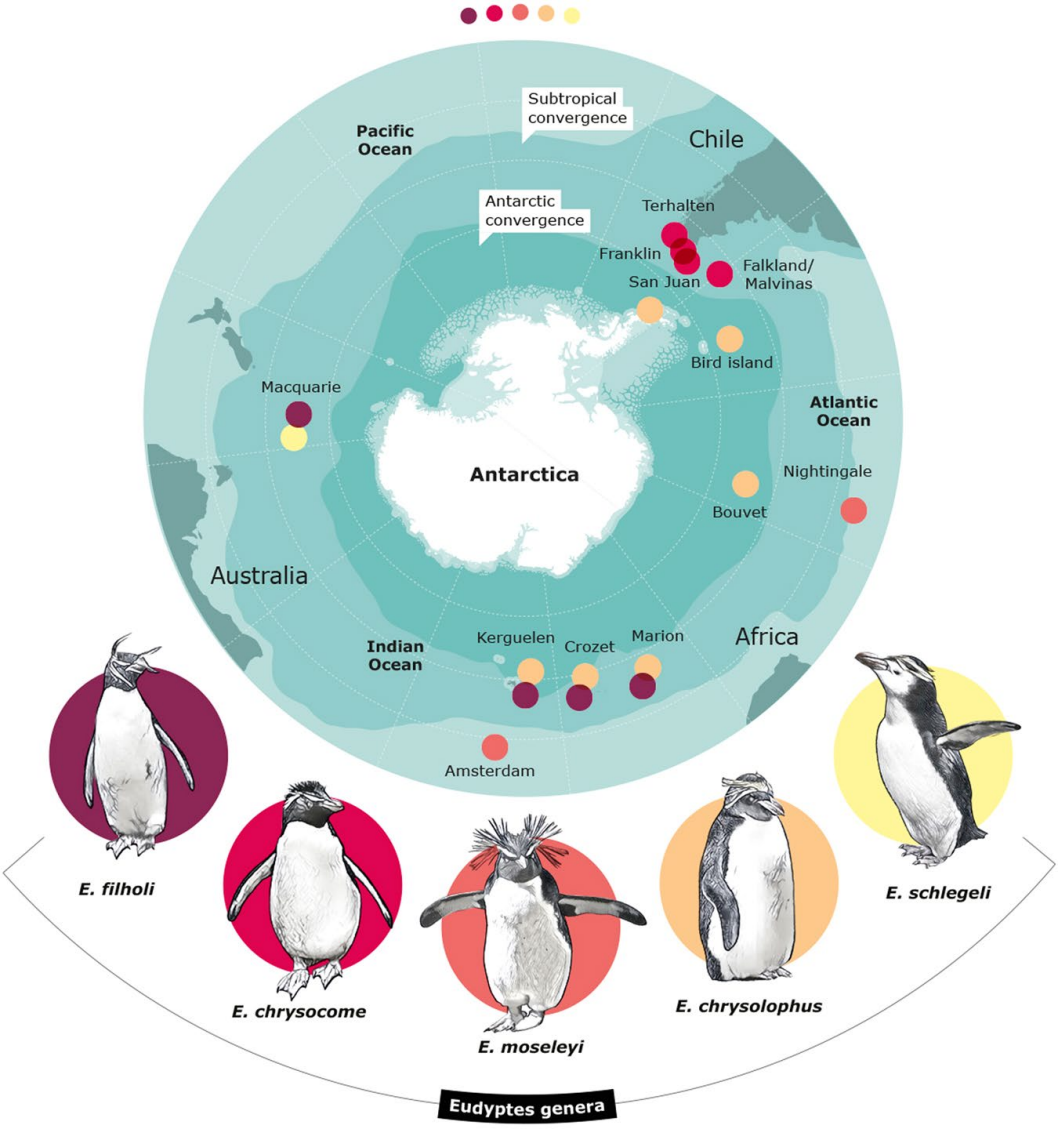
Sample locations. Locations of samples obtained from *Eudyptes* species throughout their distribution, *E. filholi* (eastern rockhopper), *E. chrysocome* (southern rockhopper), *E. moseleyi* (northern rockhopper), *E. chrysolophus* (macaroni) and E*. schlegeli* (royal). The Antarctic Convergence or Antarctic Polar Front (APF) and the Subtropical Convergence or Subtropical Front (STF) are indicated on the map.

Oceanic fronts divide the Southern Ocean into water masses with different physical characteristics, particularly in terms of temperature and salinity^1^ which have given rise to different biogeographical provinces; the physical variables are also associated with changes in species composition^2,3^. The most prominent oceanic fronts in the Southern Ocean are the Antarctic Polar Front (APF) and the Subtropical Front (STF) that separate Antarctic, subantarctic and subtropical waters^4^ (Fig. 1). The role of oceanic fronts as a barrier has been proposed for numerous marine taxa where limited genetic flow may lead to lineage differentiation and local adaptation^5,6^. Assuming that oceanic fronts are barriers for dispersal for a given species, the occurrence of populations now separated by them could be explained by previous colonization events during past glacial periods, when oceanic fronts either allowed a degree of permeability, or species moved to lower latitudes^7,8^. Different species may respond to glacial periods in different ways, e.g. becoming extinct or surviving in isolated refuges, generally promoting genetic divergences among remaining populations^9^. Also, differences in latitude and topography, for example, can affect ice coverage and timing of deglaciation between different localities in the Southern Ocean; thus, not all places were affected in the same way^10^. Therefore, the actual distribution and evolution of Antarctic and subantarctic biota are highly likely to be influenced by historical climate events and oceanographic characteristics. Hence, this past glacial history is crucial for the interpretation of the evolution of the Southern Ocean^11^.

Penguins are diving specialists distributed throughout the Southern Hemisphere. Swimming and diving facilitate both travelling and foraging but — in comparison to flying birds — may limit their migratory capabilities and distribution^12^. Currently, there are 19 recognized penguin species in six genera^13^. The crested penguins (*Eudyptes* ssp.) comprise eight species, with these different numbers reflecting taxonomic uncertainty. Their distributions range from the Antarctic Peninsula to subantarctic and subtropical waters. Four are endemic to New Zealand, subantarctic New Zealand islands and Macquarie Island (Fiordland *E. pachyrhynchus*, Snares *E. robustus;* royal *E. schlegeli* and erect-crested *E. sclateri*); in contrast, species of rockhopper (northern *E. moseleyi*, southern *E. chrysocome*, and eastern *E. filholi*) and macaroni penguins (*E. chrysolophus*) exhibit a broader distribution in the Southern Ocean^14–17^. The taxonomic status and the number of *Eudyptes* species, has been discussed repeatedly. In the 1990s, rockhopper penguins were considered to belong to one species^18^. Subsequently, based on genetic and behavioral studies they were classified as two species, the southern rockhopper, *E. chrysocome* and the northern rockhopper *E. moseleyi*^14^. In 2006, other authors suggested that rockhopper penguins should be separated into three different species, the southern *E. chrysocome*, eastern *E. filholi* and northern rockhopper, *E. moseleyi*^19^. Nevertheless, some authors still consider *E. chrysocome* and *E. filholi* a single species or consider *E. filholi* a subspecies of *E. chrysocome*^17,20^. Furthermore, royal penguins (*E. schlegeli*) — characterized by a white face phenotype — were once deemed to be a subspecies of macaroni penguins (*E. chrysolophus*), and are now considered a separate species endemic to Macquarie Island^21,22^. However, the presence of white-faced penguins has been reported from Heard, Marion, Crozet and Kerguelen islands. Thus, the question is whether they are royal penguins, an uncommon phenotype of macaroni penguin or a hybrid of royal and macaroni penguins^15^.

Macaroni (*E. chrysolophus*) and rockhopper penguins (*E. chrysocome*, *E. filholi* and *E. moseleyi*) are highly philopatric to their breeding sites^23–25^ and may also exhibit fidelity to their foraging areas, predominantly near oceanic frontal zones^26–28^ during the interbreeding period^29^. Outside the breeding season, southern, eastern and northern rockhoppers travelled up to 978 km, 3155 km and 2509 km from their colonies, respectively, and macaroni penguins travelled up to 3425 km^26,27,30,31^. These observations suggest high dispersal capabilities that could promote genetic mixing and low genetic structure among populations, even if most individuals remained philopatric^32,33^. However, oceanic fronts may also represent an efficient barrier to dispersal for these species as has been shown for other groups^5,34^. The speciation and diversification of southern (*E. chrysocome*) and eastern (*E. filholi*) from northern (*E. moseleyi*) rockhopper penguins might be explained by the presence of the STF as a biogeographical barrier coupled with a historical shift in their geographical distribution, may have resulted in the isolation of common ancestral populations^14,35^. However, recent tracking studies of rockhopper and macaroni penguins revealed that the penguins cross oceanic fronts during foraging trips in winter^26,27,36^.

Previous genetic studies using mitochondrial DNA (mtDNA, CR, ND2, cytb, 12 S, and COI) of rockhopper penguins showed differentiation between southern (Falkland Islands/Islas Malvinas), northern (Gough and Amsterdam islands) and eastern (Crozet and Kerguelen islands) populations^14,19,35^. Here, we used mtDNA (CR and COI) and nuclear markers (ODC and AK), to evaluate the congruence with previous results obtained using mtDNA. We also included DNA samples from several colonies of rockhopper penguins not previously evaluated, such as Macquarie Island and South American islands, to help clarify biogeographical patterns of eastern (*E. filholi*) and southern (*E. chrysocome*) rockhopper penguins. We expected to detect genetically structured populations within each genetic group. To date, there are no published genetic studies of macaroni penguins (*E. chrysolophus*) assessing genetic differentiation across populations. To do so, we collected several samples from macaroni penguins throughout their distributional range including populations from the Antarctic Peninsula and subantarctic islands. We also evaluated genetic differentiation between royal and macaroni penguins to assess if they are genetically isolated and correspond to truly divergent evolutionary units. Finally, white-faced penguins recently reported at Marion Island^15^ were also compared with macaroni and royal penguins to help clarify their taxonomic origin.

We hypothesize that oceanic fronts (APF and STF) represent barriers to dispersal between populations of these species and promote genetic divergence. Therefore, we expect i) a higher differentiation among breeding colonies of macaroni penguins separated by the APF than within each biogeographical region, and ii) that STF promoted higher divergence between northern (*E. moseleyi*) and southern (*E. chrysocome*) rockhoppers than between southern and eastern (*E. filholi*) rockhopper penguins.

## Methods

A total of 302 blood samples were collected: 105 from macaroni penguins (six locations), 11 from royal penguin at Macquarie Island, 55 samples from four colonies of southern rockhopper penguins, *E. chrysocome*, 49 samples from two colonies of northern rockhopper penguins *E. moseleyi* and 78 samples from four colonies of eastern rockhopper penguins, *E. filholi*. Four blood samples were also collected from penguins with the uncommon white-faced phenotype at Marion Island (Fig. 1, Table 1).

**Table 1:** Locations, geographical position and sample size of macaroni, rockhopper and royal penguins of mtDNA (CR and COI) and nuclear (ODC and AK) markers.

Species	Location	Abr.	Geographic Position	Sample size			
				HVRI	COI	ODC	AK
*E. schlegeli*	Macquarie Is., Garden C.	MACQ	54°29′S; 158°56′E	11	14	9	10
White-faced	Marion Island	MARI^1^	46°50′S; 37°48′E	3	3	4	3
*E. chrysolophus*	Crozet Island	CROZ	45°25′S; 50°24′E	18	17	18	17
	Marion Island	MARI	46°50′S; 37°48′E	21	28	25	22
	Kerguelen Island	KERG	49°42′S; 69°46′E	14	18	20	14
	Bouvet Island	BOUV	54°25′S; 03°22′E	11	12	9	10
	Bird Island	BIRD	54°00′S; 38°02′W	5	14	13	8
	Elephant Island	ELEP	61°05′S; 55°00′W	10	12	10	9
*E. moseleyi*	Nightingale Island	NIGH	37°25′S; 12°28′W	18	19	20	18
	Amsterdam Island	AMST	37°50′S; 77°31E	26	25	29	26
*E. filholi*	Crozet Island	CROZ	46°21′S; 51°42′E	23	26	26	22
	Marion Is., Whale Bird P.	MARI	46° 57′S; 37 52′E	17	14	20	11
	Kerguelen Islands	KERG	49° 28′S; 69°57′E	21	25	24	22
	Macquarie Is., Bauer Bay	MACQ	54°33′S; 158°52′E	7	10	8	8
*E. chrysocome*	Falklands/Malvinas	FALK	51°03′S; 61°07′W	12	14	9	7
	San Juan Bay, Staten Is.	SANJ	54°43′S; 63°50′W	9	10	8	6
	Franklin Bay, Staten Is.	FRAN	54°51′S; 64°39′W	17	23	16	16
	Terhalten Island	TERH	55°26′S; 67°03′W	8	8	9	2

Capture and handling of penguins followed procedures that caused the least amount of stress for both captured individuals and surrounding colony members. Birds were captured and released at the capture site after handling. The penguins were caught by hand or with a hand-held net, and then immobilized manually as described by Wilson^37^. Blood samples were taken with 23 G or 25 G needles for adults and 26 G for juveniles from the brachial or external metatarsal vein (~0.5 mL) and stored in 96% ethanol. Fecal samples were collected from four individuals from royal penguins and stored in 96% ethanol. The methodology was approved by the Ethics, Bioethics and Biosecurity committee from the Pontificia Universidad Católica de Chile (CEBB-FAIF 01/2015) following guidelines from Biosecurity Manual from CONICYT (version 2008), from the Canadian Council on Animal Care (CCAC) and Chilean law 20380 about Animal Protection. Full permission for sampling, access to the penguin colonies and animal ethics approval were granted by the respective authority responsible for the various locations (Supplementary Table S1).

DNA was isolated from blood samples using a salt protocol following Aljanabi and Martinez^38^ with modifications described in Vianna, *et al*.^34^, and from scat samples using QIAamp DNA Stool kit (Qiagen). Primers were designed for *Eudyptes* ssp. based on mtDNA genome, for mitochondrial control region (Hypervariable Region 1: HVRI; RockCRF: 5′-TGG CTT TTC TCC AAG ACC TG-3′ and RockCRR: 5′-TGG CTC TGT GAA GAG CAA GA-3′) and the cytochrome oxidase subunit 1 for penguins (COI; Cox1sphen1F: 5′-TAG CAC ACA TCA ATG AGC-3′ and Cox1sphen1R: 5′-TCT ACG TCT ATT CCG ACT G-3′). We also amplified two nuclear introns ornithine decarboxylase intron 6 (ODCF and ODC6R) and adenylate kinase 1 (AKlongF and AKlongR) described in Dantas, *et al*.^39^. Polymerase chain reactions (PCR) were performed in 30 µl volume containing 2 µl DNA at 20 ng/µl, 1X reaction buffer, 1.5 mM MgCl2, 200 µM of each dNTP, 0.4 µM of each primer and 0.8 unit Taq DNA polymerase (Invitrogen). The PCR protocol has two phases: (1) 10 min at 95 °C, and 11 cycles of 95 °C for 15 s; a touchdown of annealing temperature at 60–50 °C for 30 s^40^, with one cycle at each annealing temperature of 1 °C interval, and 72 °C for 45 s; (2) 35 amplification cycles at 95 °C for 15 s, 50 °C for 30 s, and 72 °C for 45 s; and a final extension period of 30 min at 72 °C. The mtDNA PCR products were purified and Sanger sequenced bi-directionally in Macrogen Inc. (Seoul, South Korea).

The number of individuals successfully amplified for each genetic marker is shown in Table 1. All *Eudyptes* penguin sequences were deposited in Genbank (Supplementary Table S2). Sequences were edited using Sequencher v. 5.1 (Gene Codes, Ann Arbor, MI, USA) and aligned using ClustalX v. 2.1^41^. Polymorphic sites and haplotypes were identified by DNAsp program v. 5.0^42^. To identify haplotypes of heterozygotes in the two nuclear introns we used Phase^43^, a Bayesian approach implemented in the DNAsp. Three additional COI sequences for royal penguin from Genbank (FJ582596, FJ582597, FJ582599) were incorporated for data analysis.

### Genetic Diversity

For mtDNA HVRI, COI, AK and ODC sequences, we characterized the genetic diversity of each location for all species (Table 2). We used Arlequin v. 3.5.1.2^44^ to calculate the following summary statistics: number of polymorphic sites (*S*), haplotype number (*H*), haplotype diversity (*Hd*), nucleotide diversity (*π*) and pairwise difference (∏, average number of nucleotide differences between sequences).

**Table 2:** Genetic diversity indices and Neutrality test from mtDNA HVRI, COI and Nuclear AK and ODC sequences.

		HVRI (n = 251, 451 pb)							COI (n = 290, 846 pb)				*AK* (n = 231, 532 pb)				*ODC* (n = 277, 701 pb)			
Species	Location	H	S	Hd	π	∏	*D*	*Fs*	H	S	Hd	π	H	S	Hd	π	H	S	Hd	π
*E. schlegeli*	MACQ	11	18	1.00	0.013	5.64	−0.37	−6.45***	3	2	0.38	0.0006	14	17	0.96	0.008	5	6	0.69	0.003
White-faced^1^	MARI1	2	5	0.67	0.008	3.33	0.00	2.36	1	0	0	0	2	4	0.33	0.002	3	4	0.68	0.003
*E. chrysolophus*	CROZ	13	25	0.90	0.008	3.73	−1.93	−6.27**	5	4	0.51	0.0006	19	20	0.94	0.007	8	9	0.69	0.003
	MARI	16	34	0.97	0.012	5.20	−1.76	−7.39**	8	7	0.44	0.0005	25	17	0.97	0.006	8	8	0.65	0.003
	KERG	13	36	0.99	0.019	8.25	−1.17	−5.08*	5	4	0.48	0.0006	21	22	0.95	0.008	8	8	0.70	0.003
	BOUV	9	26	0.96	0.021	9.13	0.13	−1.10	3	2	0.44	0.0005	16	15	0.98	0.008	7	8	0.86	0.004
	BIRD	5	24	1.00	0.026	11.60	0.05	−0.14	2	2	0.26	0.0005	12	14	0.95	0.007	9	9	0.76	0.002
	ELEP	9	26	0.98	0.019	8.31	−0.46	−2.09	4	3	0.45	0.0005	14	14	0.97	0.007	8	7	0.82	0.003
*E. moseleyi*	NIGH	15	19	0.98	0.007	3.14	−1.67	−11.63***	1	0	0	0	9	8	0.81	0.003	3	2	0.10	0.0002
	AMST	16	29	0.94	0.010	4.63	−1.45	−5.96*	3	2	0.16	0.0002	8	8	0.70	0.001	4	4	0.28	0.0005
*E. filholi*	CROZ	17	27	0.96	0.015	6.70	−0.32	−6.01*	5	7	0.59	0.0024	13	13	0.88	0.003	7	7	0.57	0.002
	MARI	16	27	0.99	0.018	7.99	−0.00	−8.03**	4	4	0.69	0.0019	7	8	0.79	0.003	9	8	0.65	0.002
	KERG	14	25	0.92	0.013	5.69	−0.70	−3.94	5	8	0.68	0.0026	13	13	0.86	0.003	7	6	0.63	0.001
	MACQ	7	17	1.00	0.014	4.15	−0.68	−2.55	3	5	0.73	0.0029	4	6	0.68	0.003	8	10	0.84	0.004
*E. chrysocome*	FALK	8	15	0.89	0.007	3.20	−1.53	−2.36	5	6	0.51	0.0010	7	10	0.86	0.007	5	5	0.80	0.003
	SANJ	4	14	0.69	0.008	3.61	−1.19	0.57	3	3	0.60	0.0010	8	13	0.89	0.009	8	8	0.76	0.003
	FRAN	10	25	0.92	0.009	4.07	−1.82	−4.60*	4	4	0.25	0.0004	22	13	0.92	0.008	8	7	0.76	0.003
	TERH	6	15	0.89	0.010	4.57	−1.07	−0.67	2	2	0.29	0.0006	5	9	0.89	0.008	7	7	0.63	0.002

H the number of haplotypes, S the number of polymorphic sites, Hd the haplotype diversity, ∏ the pairwise difference and π the nucleotide diversity. D Tajima’s test (D) and *Fs*, Fu’s test. ^1^White-faced penguin from Marion could be royal or macaroni penguins with white-faces or hybrids. *p < 0.05, **p < 0.01, ***p < 0.001.

### Phylogenetic reconstruction, divergence time and species delimitation

Two different methods were employed for *Eudyptes* species delimitation, the Automatic Barcoding Gap Discovery (ABGD) method (a non-tree-based method)^45^ and Generalized Mixed Yule Coalescent (GMYC) method (a single locus, tree-based method)^46^. The ABGD method is independent of tree topology and employs a genetic distance to detect a barcoding gap between candidate species based on genetic distance values that are not overlapping among intra- and interspecific comparisons^45,47^. The ABGD method was performed on the online web-server (http://wwwabi.snv.jussieu.fr/public/abgd/) and was run with the default settings (Pmin = 0.001, Pmax = 0.1, Steps = 10, X (relative gap width) = 1.5, Nb bins = 20). The mtDNA HVRI *Eudyptes* sequences alignment (without outgroup) was used to compute a matrix of pairwise distances using K2P distance. The GMYC method^46^ was implemented in R. This method is based on an ultrametric phylogenetic tree such as calibrated by molecular clock using dissimilarities of branching rates to infer species boundaries, differentiating species divergence following a Yule process and neutral coalescent events.

**Figure 2:**
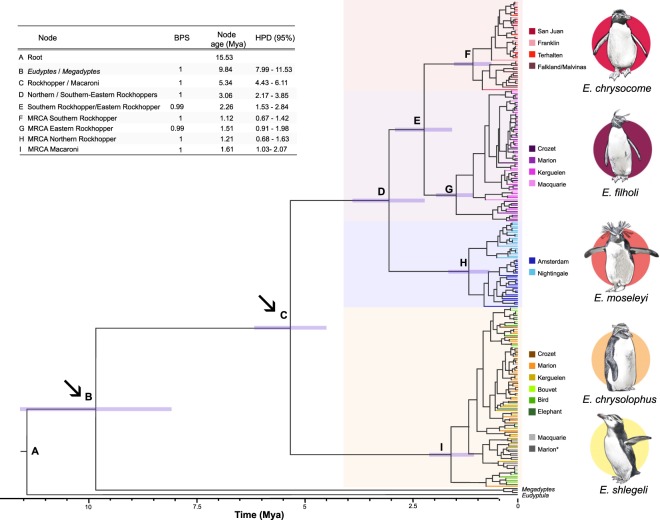
Phylogenetic reconstruction of all sampled populations of *Eudyptes* penguins. Bayesian phylogenetic tree constructed for mtDNA HVRI. Letters are represented in the table as the Bayesian posterior probabilities (BPS), divergence time in Mya and highest posterior density (HPD) in Mya. The arrows indicate the nodes calibrated using fossil record. *Represents white-faced penguins from Marion Island, and Macquarie represents royal penguins.

Bayesian phylogenetic reconstruction and divergence time estimations were implemented in the program BEAST v. 2.4.7^48^. MtDNA HVRI sequences of the yellow-eyed penguin (*Megadyptes antipodes*)^49^ and little penguin (*Eudyptula minor*) as outgroup (NC_004538) were incorporated into phylogeny (Fig. 2). The best fitting model was HKY + I + G inferred using bModeltest^50^, implemented in the software Beast2. Divergence times were calculated for mtDNA HVRI, and phylogeny was calibrated using the age of two fossil records, the *Madrynornis mirandis* (10 Mya) to the *Eudyptes/Megadyptes* split^51,52^, and *Eudyptes calauina* (5 Mya) fossil record found in Chile^53^ at *Eudyptes* split. A strict molecular clock model was applied with a prior of Yule process speciation for branching rates, and calibration prior based on normal distribution. Four independent runs were performed using 30,000,000 generations with parameters logged every 1000 steps; a burn-in of 10% trees was used. The four independent runs were combined using LogCombiner v.1.8.3 (part of the BEAST distribution). The parameter analyses for convergence and Effective Sample Size (ESS) were assessed using Tracer v. 1.6^54^. Finally, Tree annotator v. 2.4.7 was used to create a consensus tree, and FigTree v1.4.2^55^ was used to visualize the tree.

Multilocus bayesian phylogenetic reconstruction with nuclear (ODC and AK) and mtDNA (HVRI and COI) were performed in MrBayes^56,57^ and little penguin (*Eudyptula minor*) was incorporated as outgroup. The best fitting model for each marker data set was inferred using Jmodeltest 2^58,59^ and selection was made based on Akaike information criterion (AK = TVM + I + G; ODC = TrN + I; R1 = TIM1 + I + G and COI = TPM2uf + G). All four markers were concatenated into a 2532 pb alignment (HVRI = 451 pb, COI = 846 pb, AK = 532 pb, ODC = 701 pb). Two runs were performed using 10,000,000 generations, 4 chains and a burn-in of 25%.

### Phylogeographical data analyses

Genetic structure analyses were performed separately for a) rockhopper penguins (*E. chrysocome*, *E. filholi* and *E. moseleyi*), and b) macaroni royal and white-faced penguins. To evaluate genetic structure at a broader scale, we performed a Bayesian Analysis of Population Structure v. 5.4^60^ using HVRI and AK sequences. BAPS estimate genetic substructure by clustering sampled populations into groups. We performed 10 independent runs for the BAPS analysis; the resulting partitions were averaged based on their plotted posterior probabilities. To evaluate the divergence between royal and macaroni penguins, and the efficiency of the APF and STF as physical barrier, we performed Analyses of Molecular Variance (AMOVA) based on pairwise *ɸ*_*st*_. The AMOVA groups were defined for different analyses as: i) royal and macaroni; ii) macaroni penguins north (Kerguelen, Crozet and Marion islands) and south (Elephant, Bird, Bouvet islands) of the APF; iii) rockhopper penguins (*E. moseleyi*) north and (*E. chrysocome* and *E. filholi*) south of the STF.

Pairwise *F*_*st*_ and *ɸ*_*st*_ were calculated for mtDNA HVRI and AK (Supplementary Figs S1 and S3; Tables S3–S6) among locations for each species using Arlequin v. 3.5.1.2^44^ with R software incorporated. Statistical significance of the estimates was calculated realizing 10,000 permutations. The p-value for pairwise *F*_*st*_ and *ɸ*_*st*_ between populations was corrected using a false discovery rate correction^61^. We considered significant results when P < 0.05.

**Figure 3:**
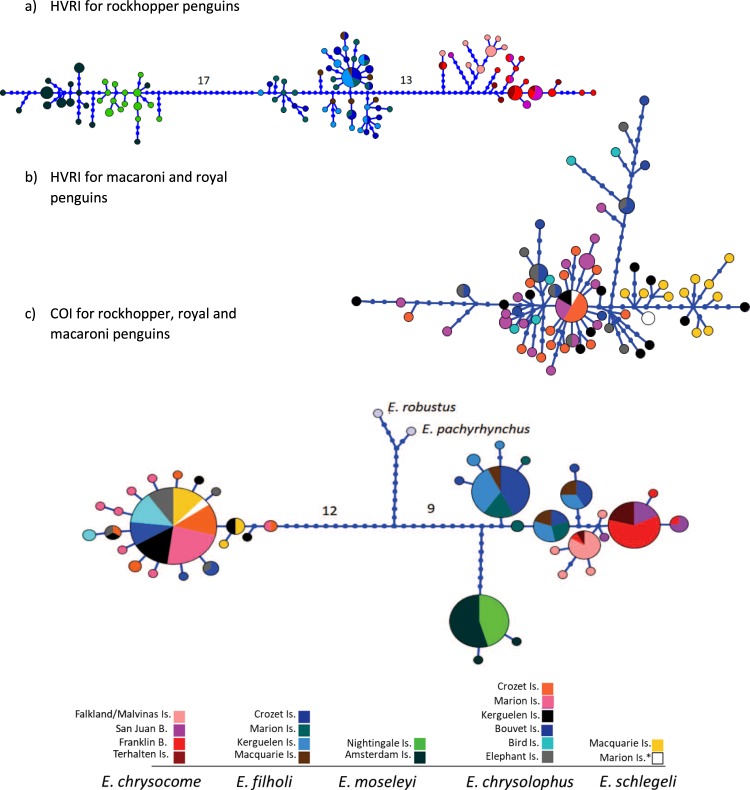
MtDNA Haplotype network for rockhopper, macaroni and royal penguins. Networks for (**a**) mtDNA HVRI in rockhopper penguins (*E. moseleyi*, *E. chrysocome* and *E. filholi*), (**b**) mtDNA HVRI in macaroni (*E. chrysolophus*) and royal penguins (*E. schlegeli*) and (**c**) COI for all species. *corresponds to white-faced penguins.

Relationships between haplotypes and their frequencies at the different locations were examined by a network based on Neighbor-joining tree^62^ in Mega v.7.0.26 for each marker in all *Eudyptes* penguins. The best substitution model was selected for each marker based on Bayesian information criterion (BIC), K2 + G (G = 0.08) for MtDNA HVRI in macaroni and royal penguins, K2 + G (G = 0.12) for MtDNA HVRI in rockhopper penguins, TN93 + G (G = 0.13) for COI of all *Eudyptes* species in this study and sequences of *Snares* and Fiordland penguins from Genbank (EU525346 and EU525344) and T92 + G (G = 0.05) for AK and ODC including all *Eudyptes* species (Fig. 3). Neighbor-joining trees were then used to construct a median-joining network (MJN) in Haploviewer Software^63^.

### Demographic history

We calculated Tajima’s *D* and Fu’s Fs indices and assessed deviation from mutation-drift equilibrium for each studied location for all species using Arlequin^44^, for all four markers with 10000 bootstrap replicates to assess significance (Table 2, Supplementary Table S7). The P-value was corrected using a false discovery rate correction^61^. We considered significant results when P < 0.05 (Supplementary Table S8).

Demographic analyses were performed using BEAST2 and Tracer v. 1.6. The Coalescent Bayesian Skyline was performed using mtDNA HVRI sequences for each genetic group for macaroni penguins, based on BAPs results. The best nucleotide substitution model was selected using the Bayesian model test package bModeltest^50^ for BEAST2 (Table S9). Analyses were run for 50 million iterations, sampling every 10,000 steps, 10% of burning, relaxed Clock Log Normal. The mutation rate assumed was 0.55 substitution/site/Mya for mtDNA HVRI based on pedigree analysis for Adélie penguins (*P. adeliae*)^64^.

## Results

### Genetic diversity

All locations and species showed high haplotype diversity (Table 2). MtDNA COI was the marker with lowest genetic diversity for all locations and species. Northern rockhopper (*E. moseleyi*) penguins exhibited the lowest haplotype and nucleotide diversity for COI, AK, ODC, and very low for HVRI for both locations compared with all other species; southern rockhoppers (*E. chrysocome*) showed the highest genetic diversity. For all markers, genetic diversity among macaroni penguins was generally highest for the southernmost locations (Bouvet, Elephant, and Bird islands).

### Divergence time and species delimitation

The Bayesian phylogenetic reconstruction based on mtDNA HVRI and the clades with high Bayesian posterior probabilities (BPS) for each node (range 0.99–1, Fig. 2) support the reciprocal monophyly and the genetic divergence (around 5.34 Mya) of macaroni and all three species of rockhopper penguins. *E. moseleyi* is a sister clade from the monophyletic group of *E. chrysocome* and *E. filholi* diverging around 3.06 Mya, followed by a more recent separation (2.26 Mya) between *E. chrysocome* and *E. filholi* (Fig. 2). Additionally, phylogenetic reconstructions of northern rockhopper, *E. moseleyi*, penguins exhibited two clades (BPS = 1), one for Amsterdam Island and the other for Nightingale Island (Tristan da Cunha archipelago). Eastern rockhoppers exhibited a single clade composed of individuals from Kerguelen, Crozet, Marion and Macquarie islands. Southern rockhoppers *E. chrysocome*, showed two clades, a mixed clade between locations in southern Patagonia, but excluding individuals from the Falkland Islands/Islas Malvinas, and the other for Falkland Island with few individuals from southern Patagonia at the clade. For macaroni, royal and whited-faced penguins the phylogenetic reconstruction did not reveal any lineage restricted to a specific geographic area. The multilocus phylogenetic reconstruction are consistent with mtDNA phylogeny supporting the reciprocal monophyly of rockhopper penguins and macaroni-royal penguins. Although the monophyly of *E. moseleyi* is supported, the unresolved branch owing to the presence of a polytomy in the clade with low posterior support value (BPS = 0.88) composed by *E. chrysocome* and *E. filholi*. However*, E. chrysocome* is grouped in a different clade than *E. filholi* (Supplementary Fig. S3).

**Figure 4:**
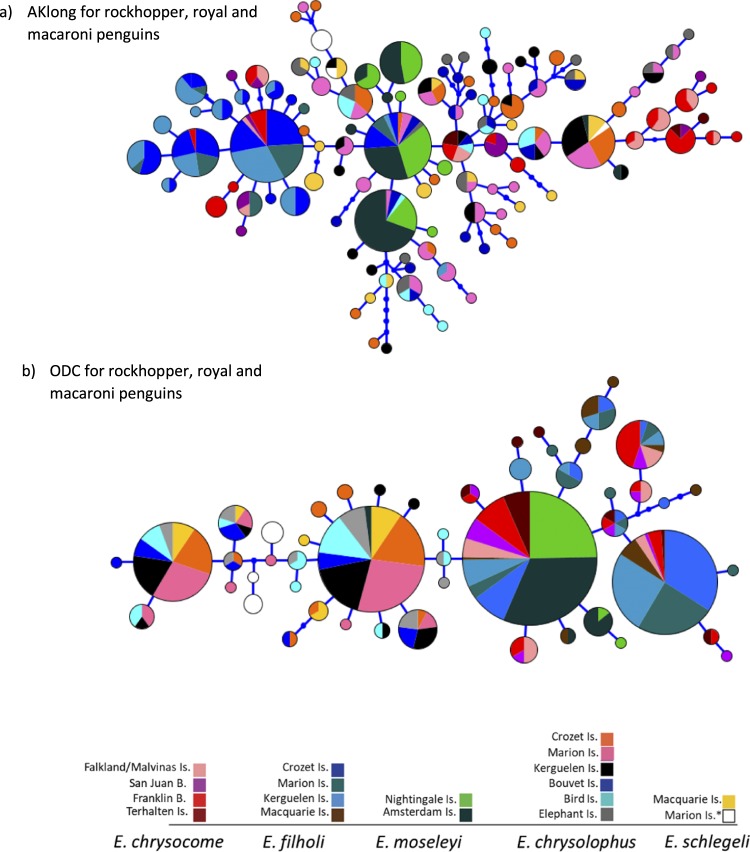
Nuclear Haplotype network for rockhopper, macaroni and royal penguins. Networks for (**a**) AKlong for rockhopper (*E. moseleyi*, *E. chrysocome* and *E. filholi*), royal (*E. schlegeli*) and macaroni (*E. chrysolophus*) penguins and (**b**) ODC for rockhopper (*E. moseleyi*, *E. chrysocome* and *E. filholi*), royal (*E. schlegeli*) and macaroni (*E. chrysolophus*) penguins. *corresponds to white-faced penguins.

COI network showed a star-like topology for macaroni and royal penguins with a common haplotype shared among all locations. MtDNA HVRI network also suggested a single genetic cluster with a star-like topology for macaroni penguins across their entire distribution, even considering the Antarctic and subantarctic region across the Antarctic Polar Front (Fig. 3). In comparison, all royal penguins from Macquarie Island (N = 11) are grouped together with white-faced penguins from Marion Island (N = 2), and some macaroni penguins from Kerguelen (N = 4). Moreover, the white-faced penguins from Marion Island exhibited two haplotypes, one belonging to the royal penguin’s haplogroup while the other corresponded to the dominant haplotype shared among macaroni penguins from Kerguelen, Crozet and Marion colonies. Among rockhopper penguins, there was a clear separation of the northern rockhopper *E. moseleyi*, from the southern *E. chrysocome* and eastern *E. filholi* rockhoppers, but a lower differentiation between the latter two species. Both COI and HVRI markers showed three divergent clusters: 1) *E. moseleyi* from Nightingale and Amsterdam islands; 2) *E. filholi* from Crozet, Marion, Kerguelen and Macquarie islands and 3) *E. chrysocome* from Terhalten Island in Chile and the two locations in Argentina at Staten Island and Falkland Islands /Islas Malvinas (Fig. 3). Moreover, *E. moseleyi* populations of Nightingale and Amsterdam were separated in two closely related haplogroups. The nuclear markers (Fig. 4) did not show a clear divergence for macaroni, royal or rockhopper penguins from different locations, nor between species. AK, the more diverse nuclear marker, did not distinguish between species and some haplotypes are shared by either macaroni, royal or rockhopper penguins. In contrast, ODC separated at least rockhopper penguins from macaroni and royal penguins. Nuclear DNA was less powerful than mtDNA at detecting phylogeographical structure possibly owing to the slower mutational rates, and also because their effective population size is four times greater than mtDNA markers and, as result, they are less affected by genetic drift. Nevertheless, results of nuclear DNA are congruent with mtDNA when they were able to detect phylogeographical structure.

Two distinct methods were employed for species delimitation. Both ABGD and GMYC method recovered the existence of four groups: macaroni (*E. chrysolophus*) + royal (*E. schlegeli*) penguins as one species, and all three rockhopper penguins (*E. moseleyi, E. chrysocome* and *E. filholi*). The ABGD analysis recovered a total of seven partitions, with partitions 1 to 5 supporting the four species with prior maximal intraspecific distances (P) ranging from 0.001 to 0.007. The GMYC delimitation model suggest five clusters and six entities (including the two species incorporated as outgroups), supporting the four *Eudyptes* species with high probabilities (confidence interval [CI] = 5–27, lnL of null model = 1034.239, ML of GMYC model = 1049.969, P = 1.47e-07***) and the threshold time of 1.28 Mya.

### Phylogeographical data analyses

**Figure 5:**
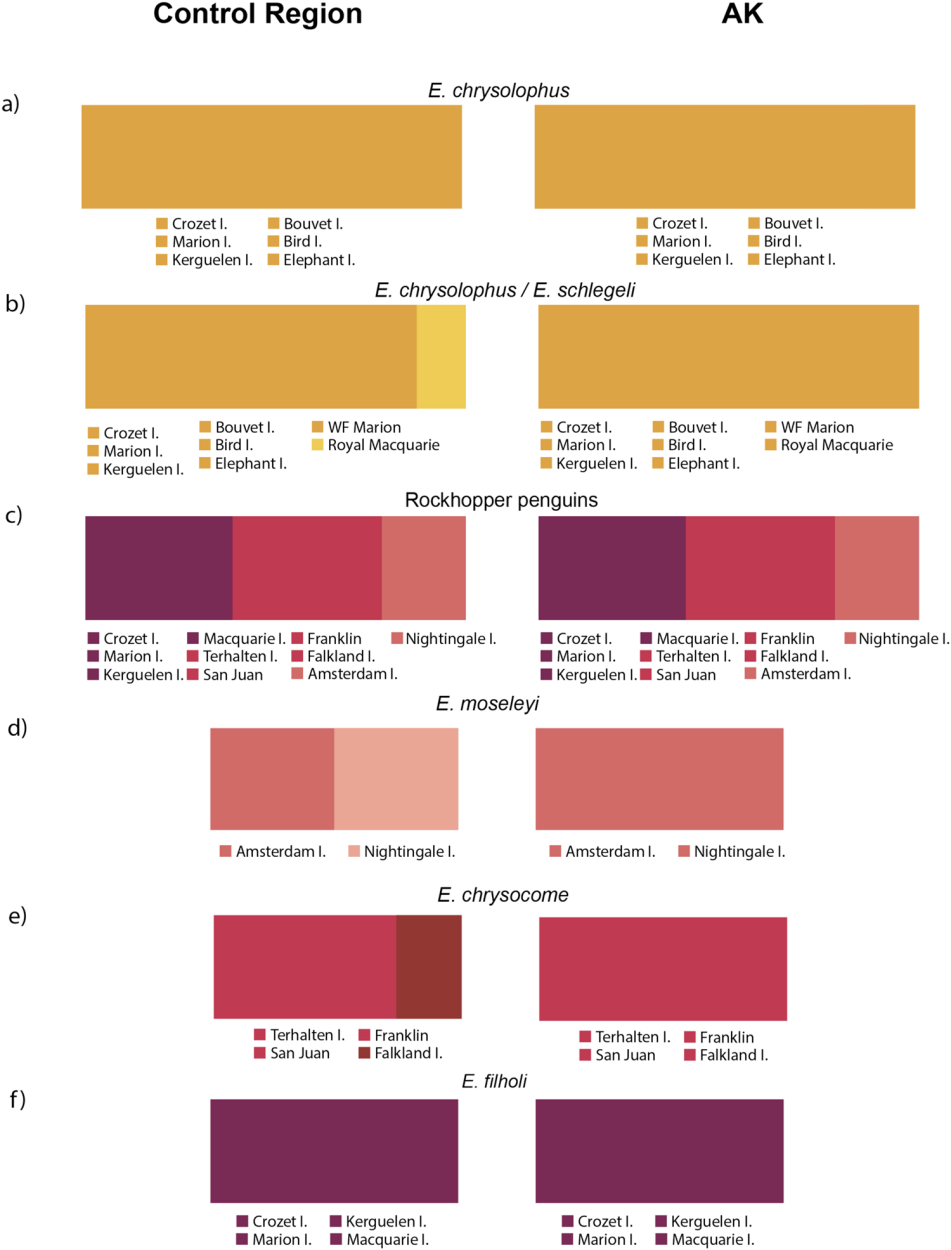
Bayesian Analysis of Population Structure (BAPS) for macaroni, royal and rockhopper penguins. Genetic clusters found by BAPS analyses in (**a**) all macaroni (*E. chrysolophus*) populations (**b**) macaroni and royal penguins (*E. schlegeli*), (**c**) all rockhopper penguins species and populations of (**d**) *E. moseleyi*, (**e**) *E. chrysocome* and (**f**) *E. filholi* separately. MARI^1^ corresponds to white-faced penguins from Marion Island.

BAPS for HVRI and AK for macaroni (*E. chrysolophus*), reveal no structure between their populations. However, when we analyze macaroni and royal (*E. schlegeli*) penguin together, BAPS for HVRI distinguished two genetic groups, while a single group was detected for the AK nuclear marker (Fig. 5). BAPS revealed three genetic groups with both HVRI and AK when rockhopper penguins (*E. moseleyi*, *E. chrysocome* and *E. filholi*) from all colonies were analyzed together, each group corresponding to one of the three proposed species: *E. moseleyi* (Amsterdam and Nightingale islands), *E. filholi* (Crozet, Marion, Kerguelen and Macquarie islands) and *E. chrysocome* (Terhalten Island, Franklin Bay, San Juan Bay and Falkland Islands/Islas Malvinas). Within species, the HVRI marker was also able to detect two groups in *E. moseleyi*, one for Amsterdam and the other for the Nightingale population, and two different groups for *E. chrysocome*, one including Terhalten, Franklin and San Juan and the other Falkland Islands/Islas Malvinas while no structure was detected among *E. filholi* populations from Marion, Crozet, Kerguelen and Macquarie islands (Fig. 5).

Highly significant *ɸ*_*st*_ values (*ɸ*_*st*_ =  0.35, P < 0.001) indicated a clear phylogeographic structure between royal penguins against all populations of macaroni penguins. AMOVA showed that the split between royal and macaroni populations explained almost 33% of total genetic variance, while < 5% was explained by differentiation among macaroni populations. When we evaluated the APF as a geographic barrier between macaroni colonies, the AMOVA showed that 11% of total genetic variance was explained by the split between the locations south (Bouvet, Bird and Elephant Island) and north of the APF (Kerguelen, Crozet and Marion Islands), while only 0.4% was explained by differences within groups. In case of rockhopper penguins the STF explained almost 60% of the total genetic variation among colonies located south and north of the STF (*ɸ*_*ct*_ = 0.58, P = 0.02). However, 26% of the genetic variance remained among colonies within northern and southern areas (*ɸ*_*sc*_ = 0.26, P < 0.001). Among group genetic variance was substantially improved when groups corresponded to the three rockhopper species (76.5%; *ɸ*_*ct*_ = 0.76, P = 0.001) but within group genetic structure was still significant (5.2%; *ɸ*_*sc*_ = 0.22, P < 0.001). Finally, best partition of genetic variance was obtained when groups corresponded to the genetic clusters detected by BAPS (80%; *ɸ*_*ct*_ = 0.8, P = 0.001). In this case, no remnant genetic structure was detected within groups (0.7%; *ɸ*_*sc*_ = 0.036, P = 0.10). Such results indicate the existence of some degree of phylogeographic structure within the rockhopper species, in particular between the Amsterdam and Nightingale populations of *E. moseleyi* (*ɸ*_*st*_ = 0.40, P < 0.001), and between Falklands/Malvinas and South American colonies (*ɸ*_*st*_ = 0.53–0.57, P < 0 0.001) in *E. chrysocome*. Values and significance of pairwise genetic structure (*F*_*st*_ and *ɸ*_*st*_) for all species and colonies are given in Supplementary material (Supplementary Figs S1 and S2; Tables S3–S6).

### Demographic History

Neutrality test performed with mtDNA HVRI marker exhibited negative and significant values only for Fu’*Fs*, especially for macaroni penguins from locations at lower latitudes including those from Marion (*Fs* = −7.39, P = 0.002), Crozet (*Fs* = −6.273, P = 0.002) and Kerguelen (*Fs* = −5.077, P = 0.02; Table 2). COI (Supplementary Tables S7 and S8) was negative and significant for Marion (*Fs* = −6.91, P = 0.000), Crozet (Fs = −2.82, P = 0.003) Kerguelen (*Fs* = −2.90, P = 0.004), and also for Elephant Island (Fs = −2.12, P = 0.007). There were no significant values for Tajima´D and Fu’*Fs* with ODC nuclear marker; however, AK was negative and Fu’*Fs* were significant for all populations of macaroni penguins (Supplementary Tables S7 and S8). Rockhopper penguins exhibited negative and significant *Fs* values for *E. moseleyi* at Nightingale (*Fs* = −11.63, P = 0.000) and Amsterdam (*Fs* = −5.96, P = 0.024), for *E. filholi* at Marion (*Fs* = −8.03, P = 0.005) and Crozet (*Fs* = −6.01, P = 0.024) and in the population of *E. chrysocome* at Franklin Bay (*Fs* = −4.60, P = 0.024; Table 2). The mtDNA COI marker was negative and the Fs significant for *E. moseleyi* at Amsterdam (Fs = −2.12, P = 0.031) and for *E. chrysocome* in Franklin Bay (*Fs* = −2.26, P = 0.031). Tajima´*D* was significant for *E. chrysocome* at the Falklands/Malvinas (D = −1.95, P = 0.008) and Franklin Bay (D = −1.88, P = 0.008). Neutrality test for rockhopper penguins performed with AK nuclear markers were not significant (Supplementary Tables S7 and S8) for all populations, whereas ODC nuclear marker exhibited negative and significant values of Fu’*Fs* in *E. moseleyi* at Nightingale (*Fs* = −2.02, P = 0.04) and *E. chrysocome* at San Juan Bay (*Fs* = −3.28, P = 0.04) and Terhalten (*Fs* = −3.47, P = 0.02).

**Figure 6:**
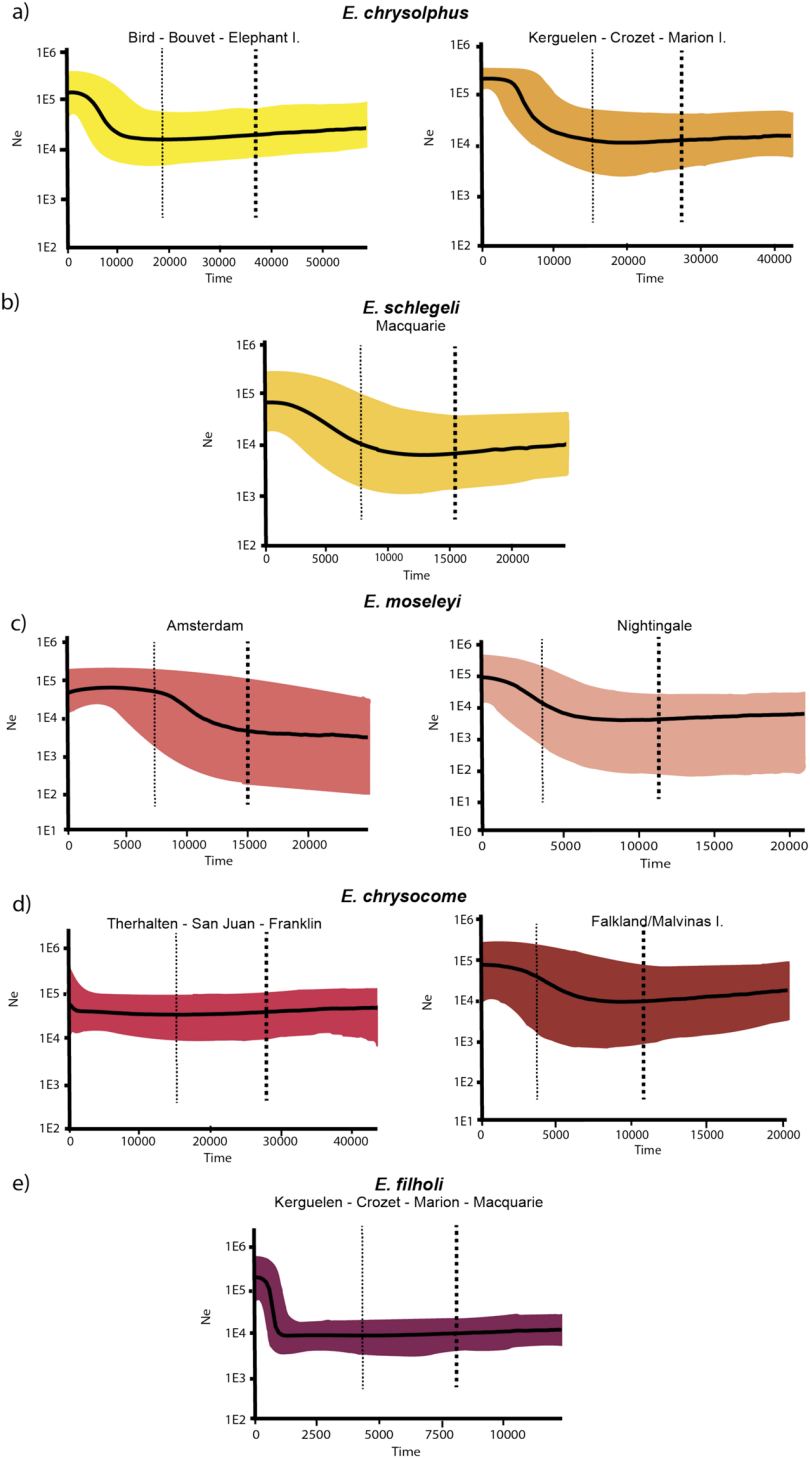
Bayesian Skyline plots for genetic groups found in *Eudyptes* penguins. Skyline Plot for each genetic group based on BAPs results using mtDNA HVRI. (**a**) Two genetic groups Bird-Bouvet-Elephant islands and Kerguelen-Crozet-Marion populations of *E. chrysolophus*, (**b**) *E. schlegeli* from Macquarie Island, (**c**) Amsterdam and Nightingale populations of *E. moseleyi*, (**d**) Terhalten, San Juan and Franklin grouped and Falklands/Malvinas separately for *E. chrysocome* and (**e**) Kerguelen, Crozet, Marion and Macquarie populations grouped together for *E. filholi*.

Skyline plots revealed population expansion for macaroni penguins from both Kerguelen-Crozet-Marion and Bird-Bouvet-Elephant island groups, around 10,000 ya (Fig. 6). Similar patterns were also observed for royal penguins from Macquarie Island. Skyline plots also suggested population expansion in colonies of *E. moseleyi*, while *E. chrysocome* from Terhalten-San Juan-Franklin exhibited a constant population size over time (Fig. 6). These results agree with the negative and significant values of Fu’s *Fs* for most populations studied in these species.

## Discussion

Oceanic fronts represent strong discontinuities in the characteristics of water masses and have been considered potential biographical barriers for marine taxa^3,65^. When oceanic fronts coincide with the boundaries of species distributions, they may be main drivers of speciation and diversification processes^34,66^. Even for species distributed across such oceanographic and biogeographic breaks, gene flow may be limited generating genetic and sometimes phylogeographic structure^67^. Based on this study, we suggest that oceanic fronts play a role in limiting gene flow among macaroni penguin populations separated by the APF, and in diversification processes for rockhopper penguins for whom the presence of the STF delimitates the occurrence of species on both sides of it; *E. moseleyi* populations are located north of STF in contrast to *E. chrysocome* and *E. filholi* populations which occur south of this front. Furthermore, particularly for populations of *E. moseleyi* and *E. chrysocome*, geographical distance may also be an important limiting factor for the dispersal between populations of these species.

Royal penguins (*E. schlegeli*) are endemic to Macquarie Island while macaroni penguins (*E. chrysolophus*) occupy subantarctic islands and islands near the Antarctic Peninsula. Morphologically, royal penguins are distinguished by their white-grey faces in contrast to the black faces of macaroni penguins. Royals were once classified as a subspecies of macaroni penguins but they are currently considered separated species^21,22^ with a recent divergence time around 2 Mya^68^. However, their present taxonomic status is debatable. Baker *et al*. (2006) performed a phylogenetic analysis based on a single individual per species and could not test whether royal penguins fulfill the phylogenetic concept of species. In our study, it appears that royal and macaroni penguins do not conform reciprocal monophyletic clades. Nuclear genes, as well as COI commonly used for species identification (Barcode), were unable to discriminate royal and macaroni penguins and may instead support the existence of a single evolutionary unit. We detected significant genetic and phylogeographic structure between both nominal species only in the case of the most variable mtDNA marker HVRI. The presence of white-faced penguins reported at Heard, Kerguelen, Crozet and Marion islands generates more confusion about the status and distribution of royal penguins^15^. In our phylogenetic and network analyses of HVRI, white-faced penguins from Marion Island exhibited two haplotypes, one belonging to the haplogroup mainly composed of royal individuals, and the other corresponded to the dominant haplotype of macaroni penguins from Kerguelen, Crozet and Marion islands. Additionally, the species delimitation methods were not able to establish royal penguins as a separate species.

Finally, our results are not fully conclusive with regard to the taxonomic status of royal and white-faced penguins. Even if most of the genetic data support that they correspond to a phenotypic variant of macaroni penguins, phylogeographic structure detected with HVRI may also indicate a recent and incipient divergence process. This question should be further investigated with genome-wide markers that should detect contemporary gene flow between royal and macaroni penguins, and also among sampled populations of macaroni penguins.

Across all sampled populations of macaroni penguins, no divergent lineages were observed (Fig. 2), suggesting an absence of historical isolation between colonies and the existence of a single evolutionary unit in this species. This is an unexpected result since breeding sites of macaroni penguins include colonies within the Antarctic Peninsula region, such as the ice-covered Elephant Island^10^ and South Georgia^69^, within the subantarctic region including Marion, Crozet and Kerguelen islands, and within the Patagonian Province such as Diego Ramirez archipelago^15^. These different regions are recognized as separated biogeographic provinces^70^, each one characterized by a wide range of varying environmental factors, including physical and chemical drivers^71^. Hence, contrasting selective pressures may have promoted local adaptations and therefore contribute to genetic divergence of taxa^72,73^. Such distribution is also shared by the gentoo penguin (*Pygoscelis papua*)^74^. Unlike macaroni penguins, lineage differentiation of gentoo penguins was found at various breeding sites^34^, which were also accompanied by differences in their reproductive period across their range^75^. Differences in reproductive chronology in this species is normally considered in terms of resource availability but it could also be related to differences in environmental factors^76^. The lack of divergent lineages and reduced population structure among macaroni penguins along an extensive gradient of environmental conditions suggests a wide tolerance to environmental factors such as temperature allowing them to survive and reproduce in different climatic conditions.

Most penguin species are thought to be highly philopatric. However, their usually large population sizes, long dispersal capabilities and dispersal events between colonies could maintain genetic homogeneity among populations^77^. In our study, BAPS analyses revealed a single genetic group among macaroni penguin populations. However, despite the lack of phylogeographic structure in macaroni penguins, some level of genetic structure has been detected among colonies, in particular between those located north and south of the APF. AMOVA identified that a substantial and significant part of genetic variation (11%) was associated with the presence of the APF, supporting our hypothesis that a reduction of gene flow was associated with this oceanographic front. High levels of genetic homogeneity between colonies are revealed in several penguin species, including the little penguin^78^, emperor (*Aptenodytes forsteri*)^79^, king (*A. patagonicus*)^33,80^, Adélie^81^, chinstrap^32,82^ and Galápagos penguins (*Spheniscus mendiculus*)^83^. King penguins displayed a similar pattern to macaroni penguins, i.e. little genetic differentiation across their wide range. However, south of the APF at South Georgia, the population exhibited significant population structure (although very low, range significant *F*_*st*_ = 0.003–0.005) with all other studied colonies north of the APF^33^. Connectivity of macaroni and king penguin colonies may be affected by the presence of the APF, although some degree of permeability may exist.

Among rockhopper penguins, differentiation into northern (*E. moseleyi*), eastern (*E. filholi*) and southern (*E. chrysocome*) rockhopper penguins is strongly supported by reciprocally monophyletic clades (Fig. 2), suggesting historical reproductive isolation between them and supporting the designation of three separate species^35^. The first divergence event corresponds to the split between *E. moseleyi*, and *E. chrysocome*-*E. filholi* species around 3.06 Mya, supporting the role of the STF as the prime driver of diversification in this genus^35^. Such a process is also evidenced by differences in morphology, nuptial calls and reproductive timing between species^14^. Divergence between southern and eastern rockhoppers was also evidenced by phylogenetic reconstructions with a calculated divergence time of 2.26 Mya and a strong BPS (0.99). de Dinechin, *et al*.^35^ suggested that vicariant events during glacial periods could be responsible for the genetic isolation of southern and eastern rockhoppers, leading to lineage differentiation. Based on our results, it is also possible that the relation of geographic distance/dispersal capabilities of eastern and southern rockhoppers could be responsible for the genetic isolation between these two groups. Two different clades were also identified within northern and southern rockhoppers. In the former, we found lineage differentiation between the Amsterdam and Nightingale populations, suggesting a historical separation of these populations. In southern rockhoppers, we found a separated clade for the Falkland Islands/Islas Malvinas population.

The higher dispersal capabilities of macaroni penguins could maintain active genetic flow among their colonies and, thus, prevent genetic isolation and lineage diversification, as seen in rockhopper penguins. Our study supports the general hypothesis that taxa with higher dispersal capabilities are associated with lower speciation rate and lineage diversification^84,85^.

A different pattern of population genetic structure was found in rockhopper species. First, BAPS analyses revealed two genetics groups within *E. moseleyi* and *E. chrysocome* but no differentiation within *E. filholi*. The northern species, *E. moseleyi*, exhibited significant genetic structure between the Nightingale and Amsterdam populations. These populations are separated from each other by >7000 km, and their dispersal capabilities could potentially be around 2000 km from their breeding colonies^26^. Therefore, a difference in genetic structure is not surprising and has also been shown in previous studies^14,35^. The haplotype distributions of these populations in the network support previous findings of long term genetic isolation between them (Fig. 3). In *E. filholi*, BAPs identified only one group which is also supported by non-significant pairwise comparison between colonies (Supplementary Fig. S2). Finally, we found two genetic groups in *E. chrysocome* identified by BAPS analyses, also supported by genetic structure (*F*_*st*_ and *ɸ*_*st*_) between Falkland Islands/Islas Malvinas and all other South American populations, but no genetic structure among San Juan-Franklin-Terhalten islands (Supplementary Fig. S2). This could be attributed to northerly-directed prevailing currents around the Falklands/Malvinas^86^; as Franklin, San Juan and Terhalten Islands are located southwest from the Falklands/Malvinas, those currents may promote isolation with limited gene flow between the Falklands/Malvinas and South America. This is also supported by the mainly northerly winter movements of this species from the Falkland Islands/Islas Malvinas^31^. Finally, compared to other penguin species, the general pattern of genetic structure in the three rockhopper species is similar to that of gentoo penguins, where population genetic structure was found even in populations separated by distances <100 km^34,87^.

The effect of glacial periods in demographic changes depends on species-specific factors such as dispersal capabilities, as well as area-specific changes in the geographic position of the sampled area (e.g. if the area was covered by ice). In the case of emperor^88,89^, king^90^ and Adélie penguins^81^, past demographic expansions are suggested as a consequence of the onset of glacial periods. Notably, these species have markedly different breeding habitats, ranging from sea-ice dependent to ice-free areas; yet they were all affected by the glacial history. This also seems to be the case for macaroni penguins. In this species, the Skyline plots suggest demographic expansions for all populations, although only Kerguelen, Crozet and Marion expansions are supported by neutrality tests. There is both terrestrial and submarine evidence of glaciation effects at Crozet, Marion, Bouvet and Elephant islands during the last glacial maximum (LGM)^10^, while ice expansion at Kerguelen and Bird islands was possibly less than at the other locations^10^. Dates of expansions (10,000 ya) suggest that population expansions were associated with the end of the LGM, as proposed for other penguin species^34,90^. More recent expansions occurred in the southernmost colonies (Bird-Bouvet-Elephant islands) compared to Kerguelen Crozet and Marion islands.

*E. filholi* is co-distributed with macaroni penguins at Marion, Crozet and Kerguelen but exhibited more recent demographic expansions around 1,000 ya (Fig. 6). Differences between species in response to past climate change are common and attributable to factors, such as dispersal capabilities, habitat requirements and prey availability^88,89^. Macaroni penguins breed in a wide range of habitat types, including the still glaciated Elephant Island^10^ suggesting that this species could breed in a glaciated scenario and be less sensitive than *E. filholi* to glacial periods. In this sense, demographic expansions of macaroni penguins could have been initiated first, even if present day temperatures were still not reached. However, this hypothesis should be investigated further.

Demographic expansion was also found for *E. chrysocome* at the Falkland Islands/Islas Malvinas around 7,000 ya, although there is little evidence for strong glaciation in this area^10^. However, other factors such as changes in marine productivity associated with glacial periods probably occurred and could have affected demographic patterns, as suggested for sea lions (*Otaria flavescens*)^91^, even if the area was not covered by ice during the LMG. Finally, demographic expansion of *E. moseleyi* at Amsterdam (~15,000 ya) and to a lesser extent at Nightingale (~7,000 ya) could also be related to the period where present day temperatures were reached after the glacial period (~10,000 years ago)^90^.

Finally, this study suggests that oceanic fronts can act as barriers for dispersal in *Eudyptes* penguins and lead to genetic isolation, although the permeability of barriers varies among species. In our study, we found three genetic units within rockhopper penguins, which are in accordance with the three species described previously. Northern rockhoppers occupy two islands, Amsterdam and Nightingale, both of which are located north of the STF. This front represents a significant biogeographic break that delimits the Southern Ocean leading to an almost complete replacement of the marine biota^2,3^. In this case, important changes in environmental and biotic characteristics associated with this boundary seem to explain the separation of these groups better than geographic distance. Amsterdam and Nightingale islands are farther apart than the colonies where eastern and southern rockhoppers breed. To a lesser extent, geographic distance could be also a relevant factor in limiting genetic flow between Amsterdam and Nightingale, as shown by the strong phylogeographical structure detected in our study. In comparison, eastern and southern rockhoppers are located within the subantartic province and are not separated by oceanic fronts. Thus, geographic distance seems to be the most relevant factor explaining their divergence. In contrast, in the case of macaroni penguins, we found a single evolutionary unit distributed over their whole extensive range including islands at the Antarctic Peninsula and subantartic provinces. However, we found weak but significant genetic structure between populations separated by the APF, suggesting it could reduce gene flow between Antarctic and subantartic populations. In our study, the designation of royal and macaroni penguin as separated species was not supported by phylogenetic and species delimitation analyses. However, a strong phylogeographical structure detected between royal and macaroni penguin populations, suggest a limited or null connectivity that may be result of geographical isolation and could reflect an incipient speciation process between them.

## Electronic supplementary material

Table S1-S9, Figure S1-S3
