# Supplementary material for: Contrasting phylogeographic pattern among Eudyptes penguins around the Southern Ocean
Source: Sci Rep. 2018 Nov 30;8:17481. doi: 10.1038/s41598-018-35975-3 (PMC6269470; doi:10.1038/s41598-018-35975-3)
Supplement: Supplementary file 1 — Table S1-S9, Figure S1-S3 [file 41598_2018_35975_MOESM1_ESM.docx]

**Contrasting phylogeographic pattern among *Eudyptes* penguins around the Southern Ocean**

Frugone, M.J.^1,2^, Lowther, A.^3^, Noll, D^1,2^, Ramos, B.^1^, Pistorius, P.^4^, Dantas, G.P.M.^5^, Petry. M.V.^6^, Bonadonna F.^7^, Steinfurth, A.^8,9^, Polanowski, A.^10^, Raya Rey, A.^11,12^, Lois, N. A.^11,13^, Pütz, K.^14^, Trathan, P.^15^, Wienecke, B.^10^, Poulin, E.^2^, Vianna, J.A.^1*^

1-Pontificia Universidad Católica de Chile, Departamento de Ecosistemas y Medio Ambiente, Vicuña Mackenna 4860, Macul, Santiago, Chile

2- Instituto de Ecología y Biodiversidad, Departamento de Ciencias Ecológicas, Universidad de Chile, Santiago, Chile

3- Norwegian Polar Institute, Tromsø, Norway, N-9297

4- DST/NRF Centre of Excellence at the Percy FitzPatrick Institute for African Ornithology, Department of Zoology, Nelson Mandela University, Port Elizabeth, 6031, South Africa

5- Pontificia Universidade Católica de Minas Gerais, PPG in Vertebrate Zoology, Belo Horizonte, Brazil

6- Universidade do Vale do Rio dos Sinos, Laboratório de Ornitologia e Animais Marinhos, Av. Unisinos, 950, São Leopoldo, RS, Brazil

7- CEFE UMR 5175, CNRS, Université de Montpellier, Université Paul-Valéry Montpellier, EPHE, 1919 route de Mende, 34293 Montpellier cedex 5, France

8- University of Cape Town, DST-NRF Centre of Excellence, Percy FitzPatrick Institute of African Ornithology, Rondebosch 7700, Cape Town, South Africa

9- RSPB Centre for Conservation Science, Royal Society for the Protection of Birds, David Attenborough Building, Pembroke Street, Cambridge, Cambridgeshire, CB2 3QZ, United Kingdom (current address)

10- Australian Antarctic Division, 203 Channel Highway Kingston, Tasmania 7050, Australia

11- Centro Austral de Investigaciones Científicas – Consejo Nacional de Investigaciones Científicas y Técnicas (CADIC-CONICET), Bernardo Houssay 200, Ushuaia, Tierra del Fuego, Argentina

12- Instituto de Ciencias Polares, Ambiente y Recursos Naturales, Universidad Nacional de Tierra del Fuego, Yrigoyen 879, Ushuaia, Argentina

13- Laboratorio de Ecología y Comportamiento Animal. Instituto de Ecologia, Genética y Evolución de Buenos Aires – Consejo Nacional de Investigaciones Científicas y Técnicas (IEGEBA-CONICET). Universidad de Buenos Aires, Argentina

14- Antarctic Research Trust, Zürich, Am Oste-Hamme-Kanal 10, 27432 Bremervörde, Germany

15- British Antarctic Survey, High Cross, Madingley Road, Cambridge, UK CB3 0ET.

Keywords: demographic history, polar region, population structure, seabirds

Corresponding author:

Juliana A. Vianna, Departamento de Ecosistemas y Medio Ambiente, Facultad de Agronomía e Ingeniería Forestal, Pontificia Universidad Católica de Chile. Av. Vicuña Mackenna 4860, Santiago, Chile, Phone: 56-2-3547210, jvianna@uc.cl

**
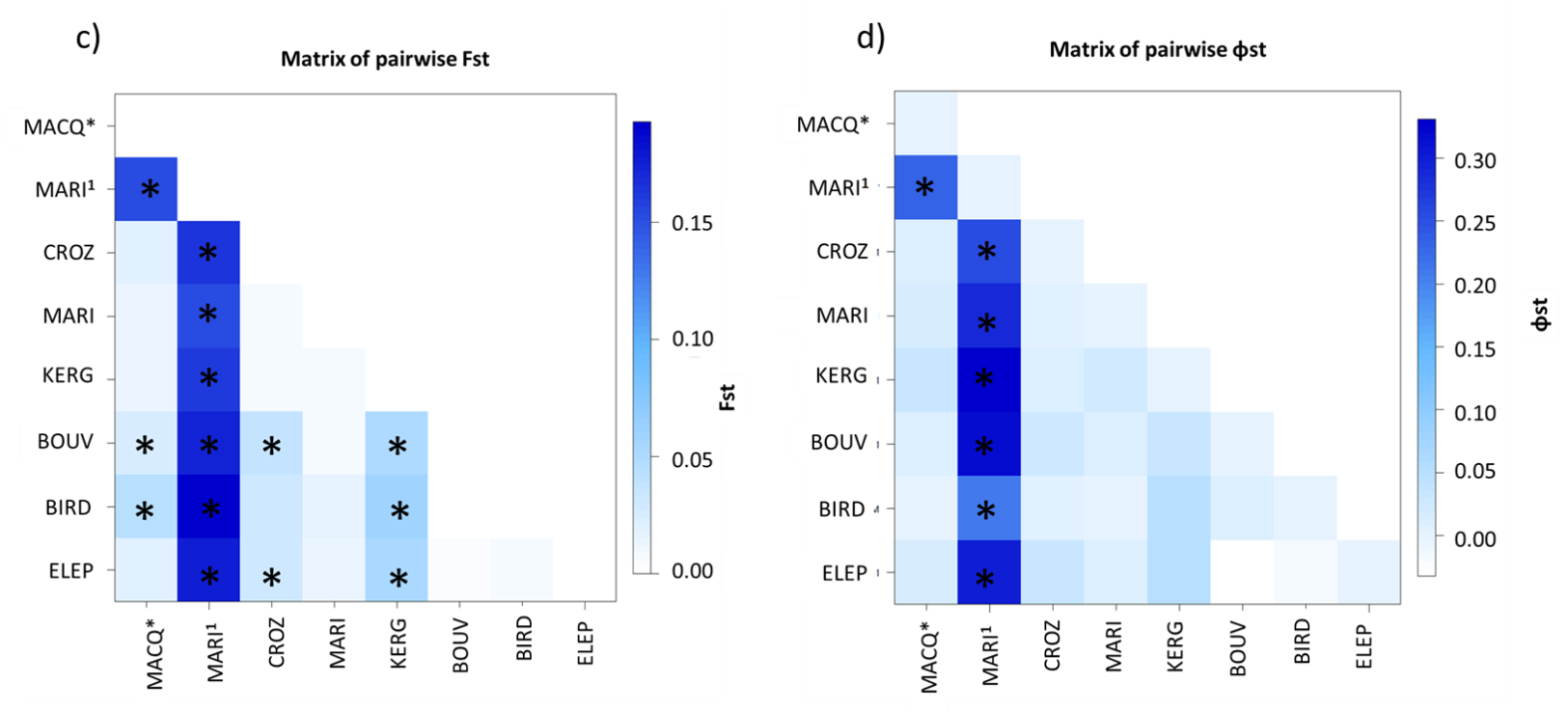

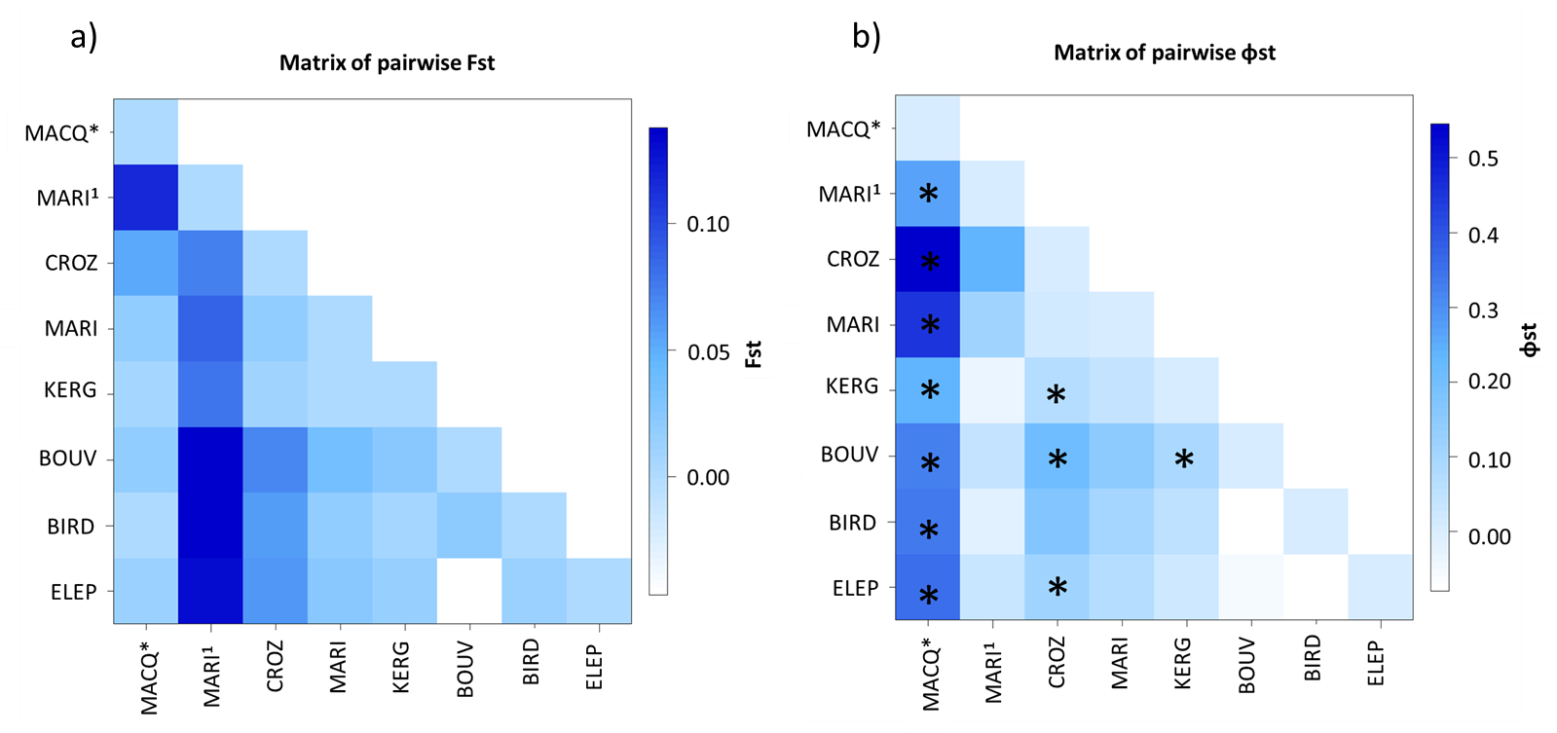
****Figure S1**. a) Pairwise FST values from mtDNA , b) pairwise ɸst from mtDNA HVR1 c) pairwise FST values from nuclear AKlong, and d) pairwise ɸst from nuclear AKlong, between the six locations for macaroni penguin, royal and for white-faced penguins. MAR1 denotes white-faced penguins from Marion and MACQ* royal penguins from Macquarie Island. *corrected P < 0.05

**Figure S2.** a) Pairwise FST values, b) pairwise ɸst for mtDNA HVR1, c) pairwise FST values from nuclear AKlong , and d) Pairwise ɸst from nuclear AKlong, of the 10 locations of rockhopper penguins. *corrected P < 0.05.


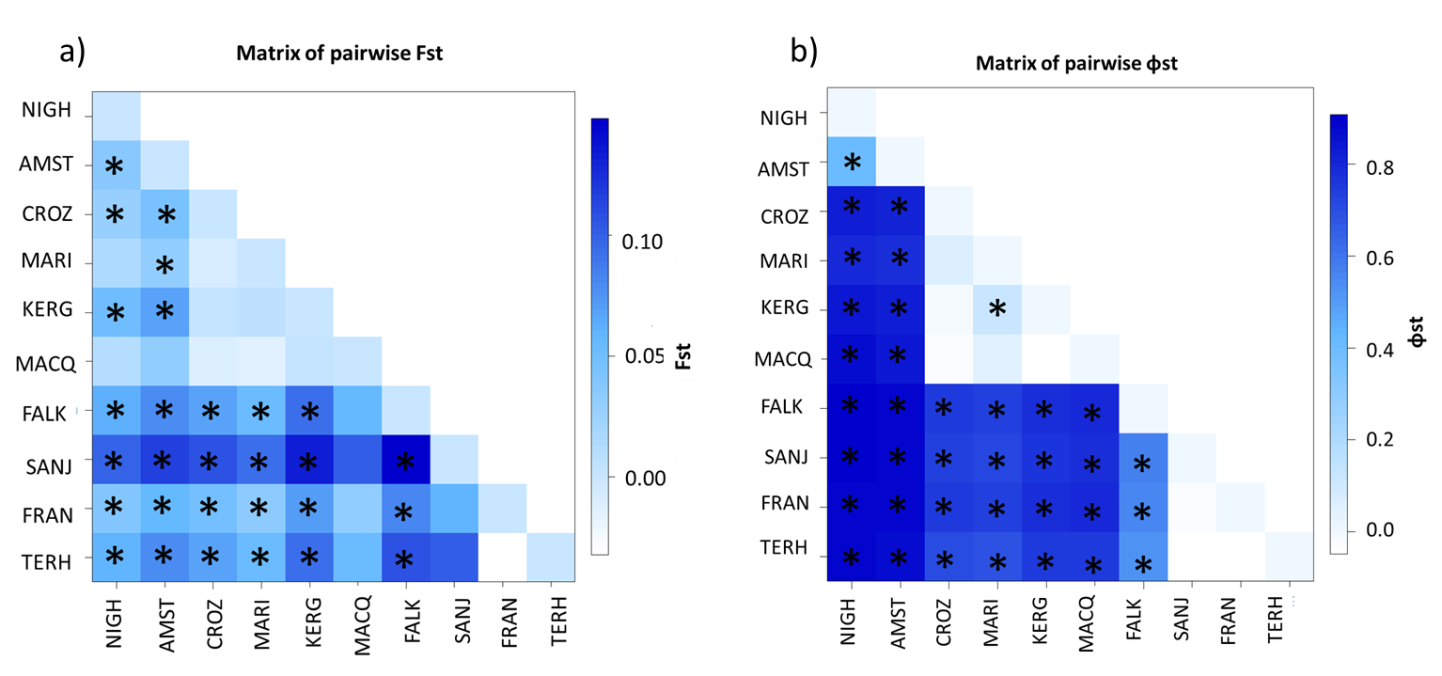

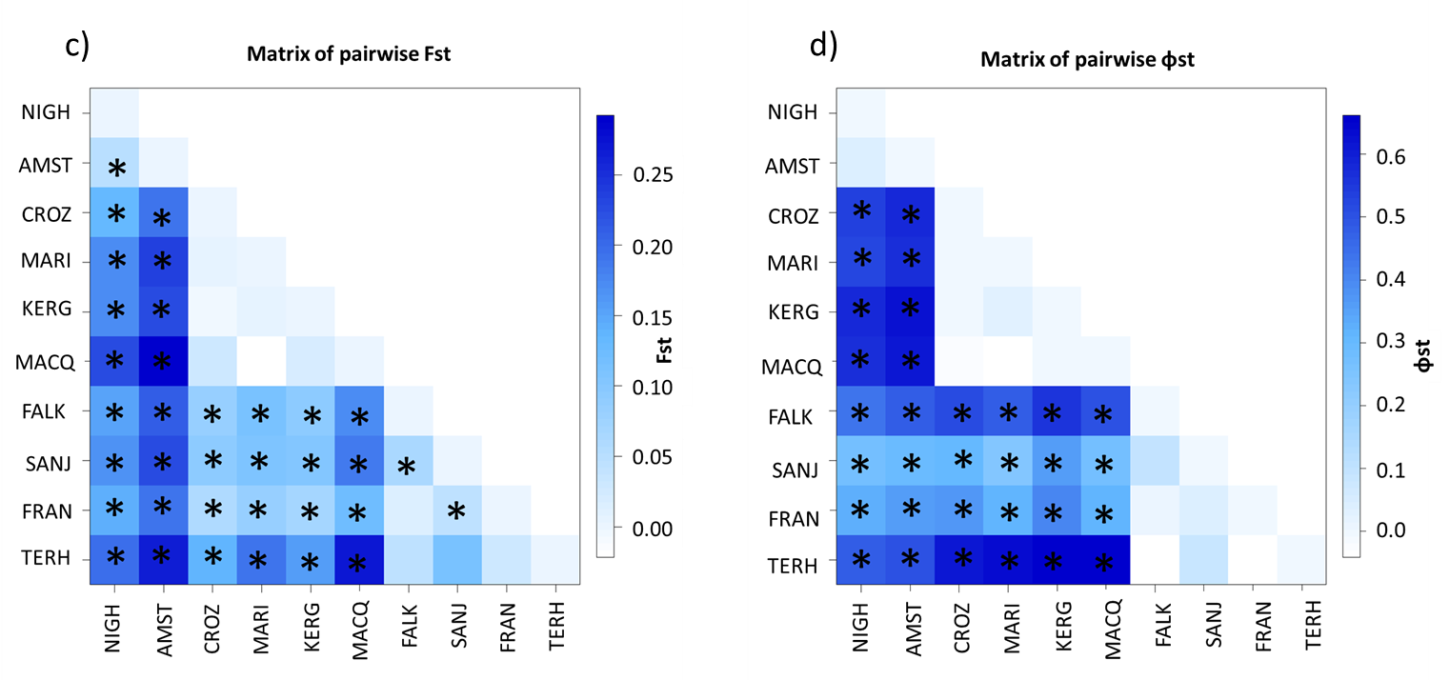


| **Specie** | **Location** | **Permision number** | **Institution** | **Provided to** |
| --- | --- | --- | --- | --- |
| *E. schlegeli* | Macquarie Is., Garden C. | TFA 09198; 431 | Department of primary industries, parks, water and environment, Tasmania | Polanowski, A; Wienecke, B. |
| White-faced | Marion Island | A14-SCI-ZOO-012 | Nelson Mandela University Research Ethics Committee | Pistorius, P. |
| *E. chrysolophus* | Crozet Island | APAFIS#9497-2017100417123472 v2 | Ministry of research; Comite d'Ethique pour l'Expérimentation Animale Languedoc Roussillon | Bonadoda, F. |
|  | Marion Island | A14-SCI-ZOO-012 | Nelson Mandela University Research Ethics Committee | Pistorius, P. |
|  | Kerguelen Island | APAFIS#9497-2017100417123472 v2 | Ministry of research; Comite d'Ethique pour l'Expérimentation Animale Languedoc Roussillon | Bonadoda, F. |
|  | Bouvet Island | 2014/230385/7001 | Mattilsynet | Lowther, A. |
|  | Bird Island | Authorized by the institution | Universty of Cambridge and British Antarctic Survey Animal Ethics and Welfare Committee; Government of South Georgia & South Sandwich Islands | Trathan, P. |
|  | Elephant Island | PPECEUA04.2017, PROANTAR nov. 2010 | UNISINOS, Progama antártico Brasileiro | Petry, M.V. |
| *E. moseleyi* | Nightingale Island | 2013/V16/AS. | Tristan government | Steinfurth, A. |
|  | Amsterdam Island | APAFIS#9497-2017100417123472 v2 | Ministry of research; Comite d'Ethique pour l'Expérimentation Animale Languedoc Roussillon | Bonadoda, F. |
| *E. filholi* | Crozet Island | APAFIS#9497-2017100417123472 v2 | Ministry of research; Comite d'Ethique pour l'Expérimentation Animale Languedoc Roussillon | Bonadoda, F. |
|  | Marion Is., Whale Bird P. | A14-SCI-ZOO-012 | Nelson Mandela University Research Ethics Committee | Pistorius, P. |
|  | Kerguelen Islands | APAFIS#9497-2017100417123472 v2 | Ministry of research; Comite d'Ethique pour l'Expérimentation Animale Languedoc Roussillon | Bonadoda, F. |
|  | Macquarie Is., Bauer Bay | TFA 09198; 431 | Department of primary industries, parks, water and environment, Tasmania | Polanowski, A; Wienecke, B. |
| *E. chrysocome* | Falklands/Malvinas | R15/2016 | Falkland Islands Government | Klemens Putz |
|  | San Juan Bay, Staten Is. | SAyDS 0781-14 | Secretaria de Ambiente y Desarrollo Sustentable de Tierra del Fuego | Andrea Raya |
|  | Franklin Bay, Staten Is. | SAyDS 0781-14 | Secretaria de Ambiente y Desarrollo Sustentable de Tierra del Fuego | Andrea Raya |
|  | Terhalten Island | 2086-2014 | Subsecretara de pesca y acuicultura, Chile; Pontificia universidad Católica de Chile | Juliana Vianna |

**Table S1.** Permission for sampling and animal ethics approval by the respective authority at each location.

**Table S2**.- Genbank accession numbers for all Species and colonies.

| **Genbank No** | **Location** | **Mitochondrial** | | **Nuclear marker** | | | |
| --- | --- | --- | --- | --- | --- | --- | --- |
|  |  | **CR** | **COI** | **AK** | | **ODC** | |
| **Northern Rockhopper Penguin, *Eudyptes moseleyi*** | | | | |  |  |  |
| NNP2751 | Nightingale I. | MG739684 |  |  |  | MH474558 | MH474559 |
| NNP2752 | Nightingale I. | MG739685 | MG739944 | MG740242 | MG740243 | MH474560 | MH474561 |
| NNP2753 | Nightingale I. | MG739686 | MG739945 | MG740244 | MG740245 | MH474562 | MH474563 |
| NNP2754 | Nightingale I. |  | MG739946 | MG740246 | MG740247 | MH474564 | MH474565 |
| NNP2755 | Nightingale I. | MG739687 | MG739947 | MG740248 | MG740249 | MH474566 | MH474567 |
| NNP2756 | Nightingale I. | MG739688 | MG739948 | MG740250 | MG740251 | MH474568 | MH474569 |
| NNP2757 | Nightingale I. | MG739689 | MG739949 | MG740252 | MG740253 | MH474570 | MH474571 |
| NNP2758 | Nightingale I. | MG739690 | MG739950 | MG740254 | MG740255 | MH474572 | MH474573 |
| NNP2759 | Nightingale I. | MG739691 | MG739951 | MG740256 | MG740257 | MH474574 | MH474575 |
| NNP2760 | Nightingale I. | MG739692 | MG739952 | MG740258 | MG740259 | MH474576 | MH474577 |
| NNP2761 | Nightingale I. | MG739693 | MG739953 | MG740260 | MG740261 | MH474578 | MH474579 |
| NNP2762 | Nightingale I. | MG739694 | MG739954 | MG740262 | MG740263 | MH474580 | MH474581 |
| NNP2763 | Nightingale I. | MG739695 | MG739955 | MG740264 | MG740265 | MH474582 | MH474583 |
| NNP2764 | Nightingale I. |  |  | MG740266 | MG740267 | MH474584 | MH474585 |
| NNP2765 | Nightingale I. | MG739696 | MG739956 | MG740268 | MG740269 | MH474586 | MH474587 |
| NNP2766 | Nightingale I. | MG739697 | MG739957 | MG740270 | MG740271 | MH474588 | MH474589 |
| NNP2767 | Nightingale I. | MG739698 | MG739958 |  |  | MH474590 | MH474591 |
| NNP2768 | Nightingale I. | MG739699 | MG739959 | MG740272 | MG740273 | MH474592 | MH474593 |
| NNP2769 | Nightingale I. | MG739700 | MG739960 | MG740274 | MG740275 | MH474594 | MH474595 |
| NNP2770 | Nightingale I. | MG739701 | MG739961 | MG740276 | MG740277 | MH474596 | MH474597 |
| ANP2527 | Amsterdan I. | MG739702 | MG739919 | MG740190 | MG740191 | MH474598 | MH474599 |
| ANP2528 | Amsterdan I. | MG739703 | MG739920 | MG740192 | MG740193 | MH474600 | MH474601 |
| ANP2529 | Amsterdan I. | MG739704 | MG739921 |  |  | MH474602 | MH474603 |
| ANP2530 | Amsterdan I. | MG739705 | MG739922 | MG740194 | MG740195 | MH474604 | MH474605 |
| ANP2531 | Amsterdan I. | MG739706 | MG739923 | MG740196 | MG740197 | MH474606 | MH474607 |
| ANP2532 | Amsterdan I. | MG739707 | MG739924 | MG740198 | MG740199 | MH474608 | MH474609 |
| ANP2533 | Amsterdan I. | MG739708 |  | MG740200 | MG740201 | MH474610 | MH474611 |
| ANP2534 | Amsterdan I. | MG739709 |  | MG740202 | MG740203 | MH474612 | MH474613 |
| ANP2535 | Amsterdan I. | MG739710 | MG739925 | MG740204 | MG740205 | MH474614 | MH474615 |
| ANP2536 | Amsterdan I. | MG739711 | MG739926 | MG740206 | MG740207 | MH474616 | MH474617 |
| ANP2537 | Amsterdan I. | MG739712 | MG739927 |  |  | MH474618 | MH474619 |
| ANP2538 | Amsterdan I. |  | MG739928 | MG740208 | MG740209 | MH474620 | MH474621 |
| ANP2539 | Amsterdan I. | MG739713 | MG739929 | MG740210 | MG740211 | MH474622 | MH474623 |
| ANP2541 | Amsterdan I. | MG739714 | MG739930 | MG740212 | MG740213 | MH474624 | MH474625 |
| ANP2542 | Amsterdan I. | MG739715 | MG739931 | MG740214 | MG740215 | MH474626 | MH474627 |
| ANP2543 | Amsterdan I. | MG739716 | MG739932 | MG740216 | MG740217 | MH474628 | MH474629 |
| ANP2544 | Amsterdan I. |  |  | MG740218 | MG740219 | MH474630 | MH474631 |
| ANP2545 | Amsterdan I. | MG739717 | MG739933 | MG740220 | MG740221 | MH474632 | MH474633 |
| ANP2546 | Amsterdan I. | MG739718 | MG739934 | MG740222 | MG740223 | MH474634 | MH474635 |
| ANP2547 | Amsterdan I. | MG739719 | MG739935 | MG740224 | MG740225 | MH474636 | MH474637 |
| ANP2548 | Amsterdan I. | MG739720 | MG739936 | MG740226 | MG740227 | MH474638 | MH474639 |
| ANP2549 | Amsterdan I. |  | MG739937 |  |  | MH474640 | MH474641 |
| ANP2550 | Amsterdan I. | MG739721 | MG739938 | MG740228 | MG740229 | MH474642 | MH474643 |
| ANP2551 | Amsterdan I. | MG739722 | MG739939 | MG740230 | MG740231 | MH474644 | MH474645 |
| ANP2552 | Amsterdan I. | MG739723 | MG739940 | MG740232 | MG740233 | MH474646 | MH474647 |
| ANP2553 | Amsterdan I. | MG739724 |  | MG740234 | MG740235 | MH474648 | MH474649 |
| ANP2554 | Amsterdan I. | MG739725 | MG739941 | MG740236 | MG740237 | MH474650 | MH474651 |
| ANP2555 | Amsterdan I. | MG739726 | MG739942 | MG740238 | MG740239 | MH474652 | MH474653 |
| ANP2556 | Amsterdan I. | MG739727 | MG739943 | MG740240 | MG740241 | MH474654 | MH474655 |
| **Southern Rockhopper Penguin, *Eudyptes chrysocome*** | | | | |  |  |  |
| CRP2497 | Crozet Island |  | MG739967 |  |  | MH474656 | MH474657 |
| CRP2500 | Crozet Island | MG739728 | MG739981 |  |  | MH474658 | MH474659 |
| CRP2501 | Crozet Island | MG739729 | MG739971 | MG740300 | MG740301 | MH474660 | MH474661 |
| CRP2502 | Crozet Island | MG739730 | MG739965 | MG740302 | MG740303 | MH474662 | MH474663 |
| CRP2504 | Crozet Island | MG739731 | MG739964 | MG740304 | MG740305 | MH474664 | MH474665 |
| CRP2505 | Crozet Island | MG739732 | MG739962 | MG740306 | MG740307 | MH474666 | MH474667 |
| CRP2506 | Crozet Island | MG739733 | MG739963 |  |  | MH474668 | MH474669 |
| CRP2507 | Crozet Island | MG739734 | MG739983 | MG740308 | MG740309 | MH474670 | MH474671 |
| CRP2508 | Crozet Island |  | MG739966 | MG740310 | MG740311 | MH474672 | MH474673 |
| CRP2509 | Crozet Island | MG739735 | MG739985 | MG740312 | MG740313 | MH474674 | MH474675 |
| CRP2510 | Crozet Island | MG739736 | MG739976 | MG740314 | MG740315 | MH474676 | MH474677 |
| CRP2511 | Crozet Island |  | MG739980 | MG740316 | MG740317 | MH474678 | MH474679 |
| CRP2512 | Crozet Island | MG739737 | MG739987 |  |  | MH474680 | MH474681 |
| CRP2513 | Crozet Island | MG739738 | MG739975 | MG740318 | MG740319 | MH474682 | MH474683 |
| CRP2515 | Crozet Island | MG739739 | MG739973 | MG740320 | MG740321 | MH474684 | MH474685 |
| CRP2516 | Crozet Island | MG739740 | MG739970 | MG740322 | MG740323 | MH474686 | MH474687 |
| CRP2517 | Crozet Island | MG739741 | MG739969 | MG740324 | MG740325 | MH474688 | MH474689 |
| CRP2518 | Crozet Island | MG739742 | MG739968 | MG740326 | MG740327 | MH474690 | MH474691 |
| CRP2519 | Crozet Island | MG739743 | MG739972 | MG740328 | MG740329 | MH474692 | MH474693 |
| CRP2520 | Crozet Island | MG739744 | MG739974 | MG740330 | MG740331 | MH474694 | MH474695 |
| CRP2521 | Crozet Island | MG739745 | MG739982 | MG740332 | MG740333 | MH474696 | MH474697 |
| CRP2522 | Crozet Island | MG739746 | MG739977 | MG740334 | MG740335 | MH474698 | MH474699 |
| CRP2523 | Crozet Island | MG739747 | MG739984 | MG740336 | MG740337 | MH474700 | MH474701 |
| CRP2524 | Crozet Island | MG739748 | MG739978 | MG740338 | MG740339 | MH474702 | MH474703 |
| CRP2525 | Crozet Island | MG739749 | MG739979 | MG740340 | MG740341 | MH474704 | MH474705 |
| CRP2526 | Crozet Island | MG739750 | MG739986 | MG740342 | MG740343 | MH474706 | MH474707 |
| KRP2470 | Kerguelen I. | MG739751 | MG740003 |  |  | MH474708 | MH474709 |
| KRP2471 | Kerguelen I. | MG739752 | MG740002 | MG740344 | MG740345 | MH474710 | MH474711 |
| KRP2472 | Kerguelen I. | MG739753 | MG740000 | MG740346 | MG740347 | MH474712 | MH474713 |
| KRP2473 | Kerguelen I. | MG739754 | MG739999 | MG740348 | MG740349 | MH474714 | MH474715 |
| KRP2474 | Kerguelen I. | MG739755 | MG740001 | MG740350 | MG740351 | MH474716 | MH474717 |
| KRP2475 | Kerguelen I. | MG739756 | MG740009 | MG740352 | MG740353 | MH474718 | MH474719 |
| KRP2476 | Kerguelen I. | MG739757 | MG739997 | MG740354 | MG740355 | MH474720 | MH474721 |
| KRP2479 | Kerguelen I. |  | MG739998 |  |  |  |  |
| KRP2480 | Kerguelen I. |  | MG740004 | MG740356 | MG740357 | MH474722 | MH474723 |
| KRP2481 | Kerguelen I. | MG739758 | MG739995 | MG740358 | MG740359 | MH474724 | MH474725 |
| KRP2482 | Kerguelen I. | MG739759 | MG739988 | MG740360 | MG740361 | MH474726 | MH474727 |
| KRP2483 | Kerguelen I. |  | MG739996 |  |  |  |  |
| KRP2484 | Kerguelen I. | MG739760 | MG740005 | MG740362 | MG740363 | MH474728 | MH474729 |
| KRP2485 | Kerguelen I. | MG739761 | MG740012 | MG740364 | MG740365 | MH474730 | MH474731 |
| KRP2486 | Kerguelen I. | MG739762 | MG740006 | MG740366 | MG740367 | MH474732 | MH474733 |
| KRP2487 | Kerguelen I. | MG739763 | MG739994 | MG740368 | MG740369 |  |  |
| KRP2488 | Kerguelen I. | MG739764 | MG739993 | MG740370 | MG740371 | MH474734 | MH474735 |
| KRP2489 | Kerguelen I. | MG739765 | MG740011 | MG740372 | MG740373 | MH474736 | MH474737 |
| KRP2490 | Kerguelen I. | MG739766 | MG739989 | MG740374 | MG740375 | MH474738 | MH474739 |
| KRP2491 | Kerguelen I. |  | MG739992 | MG740376 | MG740377 | MH474740 | MH474741 |
| KRP2492 | Kerguelen I. | MG739767 | MG740007 | MG740378 | MG740379 | MH474742 | MH474743 |
| KRP2493 | Kerguelen I. | MG739768 | MG740010 | MG740380 | MG740381 | MH474744 | MH474745 |
| KRP2494 | Kerguelen I. | MG739769 | MG739991 | MG740382 | MG740383 | MH474746 | MH474747 |
| KRP2495 | Kerguelen I. | MG739770 | MG740008 | MG740384 | MG740385 | MH474748 | MH474749 |
| KRP2496 | Kerguelen I. | MG739771 | MG739990 | MG740386 | MG740387 | MH474750 | MH474751 |
| MR5 | Marion I. | MG739772 | MG740022 | MG740296 | MG740299 | MH474790 | MH474791 |
| MR17 | Marion I. | MG739773 |  |  |  | MH474752 | MH474753 |
| MR28 | Marion I. | MG739774 |  |  |  | MH474754 | MH474755 |
| MR30 | Marion I. | MG739775 | MG740025 |  |  | MH474756 | MH474757 |
| MR31 | Marion I. | MG739776 |  |  |  | MH474758 | MH474759 |
| MR32 | Marion I. | MG739777 |  |  |  |  |  |
| MR33 | Marion I. |  | MG740020 | MG740278 | MG740279 | MH474760 | MH474761 |
| MR34 | Marion I. | MG739778 | MG740019 | MG740280 | MG740281 | MH474762 | MH474763 |
| MR37 | Marion I. | MG739779 | MG740013 | MG740282 | MG740283 | MH474764 | MH474765 |
| MR38 | Marion I. | MG739780 | MG740026 | MG740284 | MG740285 | MH474766 | MH474767 |
| MR39 | Marion I. | MG739781 | MG740015 | MG740286 | MG740287 | MH474768 | MH474769 |
| MR40 | Marion I. | MG739782 | MG740016 | MG740288 | MG740289 | MH474770 | MH474771 |
| MR42 | Marion I. | MG739783 | MG740017 |  |  |  |  |
| MR43 | Marion I. |  |  |  |  | MH474772 | MH474773 |
| MR44 | Marion I. |  |  |  |  | MH474774 | MH474775 |
| MR45 | Marion I. | MG739784 | MG740018 | MG740290 | MG740291 | MH474776 | MH474777 |
| MR46 | Marion I. | MG739785 | MG740024 | MG740292 | MG740293 | MH474778 | MH474779 |
| MR48 | Marion I. |  |  |  |  | MH474780 | MH474781 |
| MR49 | Marion I. | MG739786 | MG740023 | MG740294 | MG740295 | MH474782 | MH474783 |
| MR50 | Marion I. |  |  |  |  | MH474784 | MH474785 |
| MR51 | Marion I. | MG739787 | MG740021 | MG740297 | MG740298 | MH474786 | MH474787 |
| MR55 | Marion I. | MG739788 | MG740014 |  |  | MH474788 | MH474789 |
| MAQA54 | Macquarie I. | MH011372 | MH011385 | MH842085 | MH842086 | MH474792 | MH474793 |
| MAQA55 | Macquarie I. | MH011371 | MH011384 | MH842087 | MH842088 | MH474794 | MH474795 |
| MAQA56 | Macquarie I. | MH011370 | MH011383 | MH842089 | MH842090 | MH474796 | MH474797 |
| MAQA57 | Macquarie I. | MH011368 | MH011386 | MH842091 | MH842092 | MH474798 | MH474799 |
| MAQA58 | Macquarie I. | MH011366 |  | MH842093 | MH842094 | MH474800 | MH474801 |
| MAQA59 | Macquarie I. | MH011365 | MH011387 | MH842095 | MH842096 | MH474802 | MH474803 |
| MAQA60 | Macquarie I. | MH011367 | MH011388 | MH842097 | MH842098 | MH474804 | MH474805 |
| MAQA61 | Macquarie I. | MH011369 | MH011382 | MH842099 | MH842100 | MH474806 | MH474807 |
| **Eastern Rockhopper Penguin, *Eudyptes filholi*** | | | | |  |  |  |
| FR01N | Franklin Bay | MG739789 | MG740040 |  |  |  |  |
| FR02N | Franklin Bay | MG739790 | MG740041 | MG740388 | MG740389 |  |  |
| FR03N | Franklin Bay | MG739791 | MG740042 |  |  | MH474808 | MH474809 |
| FR04N | Franklin Bay | MG739792 | MG740043 | MG740390 | MG740391 | MH474810 | MH474811 |
| FR05N | Franklin Bay | MG739793 | MG740045 | MG740392 | MG740393 | MH474812 | MH474813 |
| FR06N | Franklin Bay |  | MG740046 | MG740394 | MG740395 | MH474814 | MH474815 |
| FR07N | Franklin Bay |  | MG740048 | MG740396 | MG740397 |  |  |
| FR08N | Franklin Bay | MG739794 | MG740050 | MG740398 | MG740399 |  |  |
| FR09N | Franklin Bay | MG739795 | MG740051 | MG740400 | MG740401 |  |  |
| FR10N | Franklin Bay | MG739796 | MG740052 | MG740402 | MG740403 | MH474816 | MH474817 |
| FR11N | Franklin Bay | MG739797 | MG740061 |  |  | MH474818 | MH474819 |
| FR12N | Franklin Bay |  | MG740053 | MG740404 | MG740405 | MH474820 | MH474821 |
| FR13N | Franklin Bay | MG739798 | MG740055 | MG740406 | MG740407 | MH474822 | MH474823 |
| FR14N | Franklin Bay | MG739799 | MG740056 | MG740408 | MG740409 |  |  |
| FR15N | Franklin Bay |  | MG740060 | MG740410 | MG740411 | MH474824 | MH474825 |
| FR16N | Franklin Bay | MG739800 | MG740039 |  |  | MH474826 | MH474827 |
| FR17N | Franklin Bay |  | MG740058 | MG740412 | MG740413 | MH474828 | MH474829 |
| FR18N | Franklin Bay | MG739801 | MG740059 |  |  | MH474830 | MH474831 |
| FR19N | Franklin Bay | MG739802 | MG740057 | MG740414 | MG740415 | MH474832 | MH474833 |
| FR20N | Franklin Bay | MG739803 | MG740054 |  |  | MH474834 | MH474835 |
| FR21N | Franklin Bay | MG739804 | MG740049 | MG740416 | MG740417 | MH474836 | MH474837 |
| FR22N | Franklin Bay | MG739805 | MG740047 |  |  |  |  |
| FR23N | Franklin Bay |  | MG740044 | MG740418 | MG740419 | MH474838 | MH474839 |
| SJ01N | San Juan Bay | MG739806 | MG740031 | MG740434 | MG740435 | MH474840 | MH474841 |
| SJ02N | San Juan Bay |  | MG740032 |  |  |  |  |
| SJ03N | San Juan Bay | MG739807 | MG740033 | MG740436 | MG740437 | MH474842 | MH474843 |
| SJ04N | San Juan Bay | MG739808 | MG740034 | MG740438 | MG740439 | MH474844 | MH474845 |
| SJ05N | San Juan Bay | MG739809 | MG740038 | MG740440 | MG740441 | MH474846 | MH474847 |
| SJ06N | San Juan Bay | MG739810 | MG740035 | MG740442 | MG740443 | MH474848 | MH474849 |
| SJ07N | San Juan Bay | MG739811 | MG740037 |  |  | MH474850 | MH474851 |
| SJ08N | San Juan Bay | MG739812 | MG740029 | MG740444 | MG740445 | MH474852 | MH474853 |
| SJ09N | San Juan Bay | MG739813 | MG740030 |  |  | MH474854 | MH474855 |
| SJ10N | San Juan Bay | MG739814 | MG740036 |  |  |  |  |
| TRP2198 | Terhalten I. | MG739815 |  |  |  | MH474856 | MH474857 |
| TRP2199 | Terhalten I. | MG739816 |  |  |  | MH474858 | MH474859 |
| TRP2201 | Terhalten I. | MG739817 | MG740068 |  |  | MH474860 | MH474861 |
| TRP2203 | Terhalten I. | MG739818 | MG740062 |  |  | MH474862 | MH474863 |
| TRP2204 | Terhalten I. | MG739819 | MG740067 | MG740446 | MG740447 | MH474864 | MH474865 |
| TRP2206 | Terhalten I. |  | MG740066 |  |  |  |  |
| TRP2207 | Terhalten I. | MG739820 | MG740065 | MG740448 | MG740449 | MH474866 | MH474867 |
| TRP2208 | Terhalten I. |  | MG740064 |  |  |  |  |
| TRP2209 | Terhalten I. | MG739821 | MG740069 |  |  | MH474868 | MH474869 |
| TRP2210 | Terhalten I. | MG739822 | MG740063 |  |  | MH474870 | MH474871 |
| IMA7N | Falkland I. | MG739823 | MG740070 | MG740420 | MG740421 | MH474872 | MH474873 |
| IMA8N | Falkland I. | MG739824 | MG740083 |  |  |  |  |
| IMA9N | Falkland I. | MG739825 | MG740073 | MG740422 | MG740423 | MH474874 | MH474875 |
| IMD7N | Falkland I. | MG739826 | MG740081 |  |  |  |  |
| IMD8N | Falkland I. |  | MG740082 |  |  |  |  |
| IMB7N | Falkland I. | MG739827 | MG740074 | MG740424 | MG740425 | MH474876 | MH474877 |
| IMB8N | Falkland I. | MG739828 | MG740075 | MG740426 | MG740427 | MH474878 | MH474879 |
| IMB9N | Falkland I. |  | MG740077 |  |  | MH474880 | MH474881 |
| IME7N | Falkland I. | MG739829 | MG740080 |  |  | MH474884 | MH474885 |
| IMG7N | Falkland I. | MG739830 | MG740076 | MG740432 | MG740433 |  |  |
| IMF8N | Falkland I. | MG739831 | MG740079 |  |  | MH474888 | MH474889 |
| IMF7N | Falkland I. | MG739832 | MG740071 | MG740430 | MG740431 |  |  |
| IME8N | Falkland I. | MG739833 | MG740072 |  |  | MH474886 | MH474887 |
| IMC7N | Falkland I. | MG739834 | MG740078 | MG740428 | MG740429 | MH474882 | MH474883 |
| **Macaroni penguin, *Eudyptes chrysolophus*** | | | |  |  |  |  |
| BMP2167 | Bouvet I. | MG739838 | MG740084 | MG740450 | MG740451 | MH474890 | MH474891 |
| BMP2168 | Bouvet I. | MG739835 | MG740085 | MG740452 | MG740453 | MH474892 | MH474893 |
| BMP2169 | Bouvet I. | MG739839 | MG740089 | MG740454 | MG740455 | MH474894 | MH474895 |
| BMP2172 | Bouvet I. | MG739836 | MG740090 | MG740456 | MG740457 | MH474896 | MH474897 |
| BMP2173 | Bouvet I. | MG739842 | MG740086 | MG740458 | MG740459 | MH474898 | MH474899 |
| BMP2174 | Bouvet I. | MG739837 | MG740088 | MG740460 | MG740461 | MH474900 | MH474901 |
| BMP2176 | Bouvet I. | MG739841 | MG740087 | MG740462 | MG740463 | MH474902 | MH474903 |
| BMP2177 | Bouvet I. | MG739843 | MG740091 | MG740464 | MG740465 |  |  |
| BMP2181 | Bouvet I. | MG739840 | MG740092 | MG740466 | MG740467 | MH474904 | MH474905 |
| BMP2191 | Bouvet I. | MG739845 | MG740093 |  |  |  |  |
| BMP2192 | Bouvet I. |  | MG740094 |  |  |  |  |
| BMP2196 | Bouvet I. | MG739844 | MG740095 | MG740468 | MG740469 | MH474906 | MH474907 |
| LMP2018 | Elephant I. | MG739851 | MG740106 | MG740548 | MG740549 | MH474908 | MH474909 |
| LMP2019 | Elephant I. | MG739848 | MG740104 | MG740550 | MG740551 | MH474910 | MH474911 |
| LMP2020 | Elephant I. | MG739854 | MG740105 | MG740552 | MG740553 | MH474912 | MH474913 |
| LMP2022 | Elephant I. | MG739852 | MG740103 | MG740554 | MG740555 | MH474914 | MH474915 |
| LMP2026 | Elephant I. |  | MG740102 |  |  |  |  |
| LMP2028 | Elephant I. | MG739850 | MG740101 | MG740556 | MG740557 | MH474916 | MH474917 |
| LMP2030 | Elephant I. | MG739849 | MG740100 | MG740558 | MG740559 | MH474918 | MH474919 |
| LMP2037 | Elephant I. | MG739853 | MG740099 | MG740560 | MG740561 | MH474920 | MH474921 |
| LMP2038 | Elephant I. | MG739847 | MG740107 |  |  | MH474922 | MH474923 |
| LMP2040 | Elephant I. |  | MG740098 |  |  |  |  |
| LMP2041 | Elephant I. | MG739855 | MG740097 | MG740562 | MG740563 | MH474924 | MH474925 |
| LMP2350 | Elephant I. | MG739846 | MG740096 | MG740564 | MG740565 | MH474926 | MH474927 |
| KMP2559 | Kerguelen I. | MG739861 | MG740169 | MG740520 | MG740521 | MH474928 | MH474929 |
| KMP2561 | Kerguelen I. |  | MG740181 | MG740522 | MG740523 | MH474930 | MH474931 |
| KMP2562 | Kerguelen I. | MG739859 | MG740182 | MG740524 | MG740525 | MH474932 | MH474933 |
| KMP2563 | Kerguelen I. | MG739864 | MG740180 | MG740526 | MG740527 | MH474934 | MH474935 |
| KMP2565 | Kerguelen I. | MG739862 | MG740179 | MG740528 | MG740529 | MH474936 | MH474937 |
| KMP2567 | Kerguelen I. | MG739857 | MG740178 |  |  | MH474938 | MH474939 |
| KMP2569 | Kerguelen I. |  | MG740177 | MG740530 | MG740531 | MH474940 | MH474941 |
| KMP2571 | Kerguelen I. | MG739863 | MG740176 | MG740532 | MG740533 | MH474942 | MH474943 |
| KMP2572 | Kerguelen I. |  | MG740175 | MG740534 | MG740535 | MH474944 | MH474945 |
| KMP2573 | Kerguelen I. | MG739858 | MG740174 | MG740536 | MG740537 | MH474946 | MH474947 |
| KMP2575 | Kerguelen I. | MG739856 | MG740173 | MG740538 | MG740539 | MH474948 | MH474949 |
| KMP2576 | Kerguelen I. | MG739860 | MG740167 | MG740540 | MG740541 | MH474950 | MH474951 |
| KMP2578 | Kerguelen I. | MG739866 | MG740172 |  |  | MH474952 | MH474953 |
| KMP2580 | Kerguelen I. | MG739868 | MG740168 | MG740542 | MG740543 | MH474954 | MH474955 |
| KMP2582 | Kerguelen I. |  | MG740183 |  |  | MH474956 | MH474957 |
| KMP2583 | Kerguelen I. | MG739865 | MG740171 |  |  | MH474958 | MH474959 |
| KMP2585 | Kerguelen I. | MG739869 | MG740184 | MG740544 | MG740545 | MH474960 | MH474961 |
| KMP2586 | Kerguelen I. | MG739867 | MG740170 | MG740546 | MG740547 | MH474962 | MH474963 |
| CMP2587 | Crozet Island | MG739877 | MG740126 | MG740470 | MG740471 | MH474964 | MH474965 |
| CMP2590 | Crozet Island | MG739885 | MG740127 | MG740472 | MG740473 | MH474966 | MH474967 |
| CMP2591 | Crozet Island | MG739884 | MG740138 | MG740474 | MG740475 | MH474968 | MH474969 |
| CMP2595 | Crozet Island | MG739883 | MG740128 | MG740476 | MG740477 | MH474970 | MH474971 |
| CMP2596 | Crozet Island | MG739880 | MG740122 | MG740478 | MG740479 | MH474972 | MH474973 |
| CMP2597 | Crozet Island | MG739881 | MG740137 | MG740480 | MG740481 | MH474974 | MH474975 |
| CMP2599 | Crozet Island | MG739878 | MG740136 | MG740482 | MG740483 | MH474976 | MH474977 |
| CMP2600 | Crozet Island | MG739875 | MG740135 |  |  | MH474978 | MH474979 |
| CMP2601 | Crozet Island | MG739882 | MG740134 | MG740484 | MG740485 | MH474980 | MH474981 |
| CMP2602 | Crozet Island | MG739874 | MG740123 | MG740486 | MG740487 | MH474982 | MH474983 |
| CMP2605 | Crozet Island | MG739873 | MG740124 | MG740488 | MG740489 | MH474984 | MH474985 |
| CMP2606 | Crozet Island | MG739887 | MG740133 | MG740490 | MG740491 | MH474986 | MH474987 |
| CMP2610 | Crozet Island | MG739886 | MG740132 | MG740492 | MG740493 | MH474988 | MH474989 |
| CMP2611 | Crozet Island | MG739872 | MG740131 | MG740494 | MG740495 | MH474990 | MH474991 |
| CMP2612 | Crozet Island | MG739871 | MG740125 | MG740496 | MG740497 | MH474992 | MH474993 |
| CMP2613 | Crozet Island | MG739879 | MG740130 | MG740498 | MG740499 | MH474994 | MH474995 |
| CMP2614 | Crozet Island | MG739870 | MG740129 | MG740500 | MG740501 | MH474996 | MH474997 |
| CMP2615 | Crozet Island | MG739876 |  | MG740502 | MG740503 | MH474998 | MH474999 |
| MMP2366 | Marion I. | MG739900 | MG740166 | MG740566 | MG740567 |  |  |
| MMP2371 | Marion I. |  | MG740163 | MG740568 | MG740569 | MH475000 | MH475001 |
| MMP2375 | Marion I. | MG739895 | MG740162 |  |  | MH475002 | MH475003 |
| MMP2378 | Marion I. |  | MG740161 | MG740570 | MG740571 | MH475004 | MH475005 |
| MMP2380 | Marion I. |  | MG740160 | MG740572 | MG740573 | MH475006 | MH475007 |
| MMP2384 | Marion I. | MG739889 | MG740159 |  |  | MH475008 | MH475009 |
| MMP2396 | Marion I. |  | MG740140 | MG740574 | MG740575 | MH475010 | MH475011 |
| MMP2397 | Marion I. |  | MG740158 | MG740576 | MG740577 | MH475012 | MH475013 |
| MMP2403 | Marion I. |  | MG740157 |  |  |  |  |
| MMP2406 | Marion I. | MG739896 | MG740156 | MG740578 | MG740579 | MH475014 | MH475015 |
| MMP2410 | Marion I. | MG739906 | MG740155 | MG740580 | MG740581 | MH475016 | MH475017 |
| MMP2411 | Marion I. | MG739908 | MG740154 | MG740582 | MG740583 | MH475018 | MH475019 |
| MMP2413 | Marion I. | MG739894 | MG740141 | MG740584 | MG740585 | MH475020 | MH475021 |
| MMP2416 | Marion I. | MG739903 | MG740164 | MG740586 | MG740587 | MH475022 | MH475023 |
| MMP2418 | Marion I. |  | MG740153 | MG740588 | MG740589 |  |  |
| MMP2420 | Marion I. | MG739899 | MG740165 |  |  | MH475024 | MH475025 |
| MMP2421 | Marion I. | MG739893 | MG740152 | MG740590 | MG740591 | MH475026 | MH475027 |
| MMP2422 | Marion I. | MG739890 | MG740151 | MG740592 | MG740593 | MH475028 | MH475029 |
| MMP2424 | Marion I. | MG739904 | MG740150 | MG740594 | MG740595 | MH475030 | MH475031 |
| MMP2426 | Marion I. | MG739897 | MG740149 | MG740596 | MG740597 | MH475032 | MH475033 |
| MMP2429 | Marion I. | MG739902 | MG740143 | MG740598 | MG740599 | MH475034 | MH475035 |
| MMP2430 | Marion I. | MG739888 | MG740147 |  |  | MH475036 | MH475037 |
| MMP2431 | Marion I. | MG739898 | MG740148 | MG740600 | MG740601 | MH475038 | MH475039 |
| MMP2434 | Marion I. | MG739892 | MG740146 | MG740602 | MG740603 | MH475040 | MH475041 |
| MMP2436 | Marion I. | MG739901 | MG740145 | MG740604 | MG740605 | MH475042 | MH475043 |
| MMP2439 | Marion I. | MG739905 | MG740144 |  |  | MH475044 | MH475045 |
| MMP2441 | Marion I. | MG739891 | MG740142 | MG740606 | MG740607 | MH475046 | MH475047 |
| MMP2443 | Marion I. | MG739907 | MG740139 | MG740608 | MG740609 | MH475048 | MH475049 |
| MWP2466 | Marion I.* | MG739916 | MG740189 | MG740610 | MG740611 | MH475076 | MH475077 |
| MWP2467 | Marion I.* | MG739914 | MG740188 |  |  | MH475078 | MH475079 |
| MWP2468 | Marion I.* | MG739915 | MG740187 | MG740612 | MG740613 | MH475080 | MH475081 |
| MWP2469A | Marion I.* |  |  | MG740614 | MG740615 | MH475082 | MH475083 |
| EMP2837A | Bird I. |  |  | MG740504 | MG740505 |  |  |
| EMP2838 | Bird I. |  | MG740120 |  |  | MH475050 | MH475051 |
| EMP2839 | Bird I. |  | MG740121 | MG740506 | MG740507 | MH475052 | MH475053 |
| EMP2840 | Bird I. |  | MG740117 |  |  | MH475054 | MH475055 |
| EMP2841 | Bird I. |  | MG740116 | MG740508 | MG740509 | MH475056 | MH475057 |
| EMP2842 | Bird I. | MG739909 | MG740115 | MG740510 | MG740511 | MH475058 | MH475059 |
| EMP2843 | Bird I. |  | MG740114 |  |  |  |  |
| EMP2844 | Bird I. | MG739912 | MG740113 |  |  | MH475060 | MH475061 |
| EMP2845 | Bird I. |  | MG740118 |  |  | MH475062 | MH475063 |
| EMP2846 | Bird I. |  | MG740112 |  |  | MH475064 | MH475065 |
| EMP2847 | Bird I. |  | MG740119 | MG740512 | MG740513 | MH475066 | MH475067 |
| EMP2848 | Bird I. |  | MG740111 | MG740514 | MG740515 | MH475068 | MH475069 |
| EMP2849 | Bird I. | MG739911 | MG740110 |  |  | MH475070 | MH475071 |
| EMP2850 | Bird I. | MG739910 | MG740109 | MG740516 | MG740517 | MH475072 | MH475073 |
| EMP2851 | Bird I. | MG739913 | MG740108 | MG740518 | MG740519 | MH475074 | MH475075 |
| **Royal Penguin, *Eudyptes schlegeli*** | | | |  |  |  |  |
| QSMI09303 | Macquarie I. | MG739917 | MG740185 |  |  |  |  |
| QSA03-1 | Macquarie I. | MG739918 | MG740186 | MG740616 | MG740617 | MH475100 | MH475101 |
| QRMI09753 | Macquarie I. |  | MG740027 |  |  |  |  |
| QRA34-2 | Macquarie I. |  | MG740028 |  |  |  |  |
| ROYA53 | Macquarie I. | MH011356 | MH011381 | MH842101 | MH842102 |  |  |
| ROYA62 | Macquarie I. | MH011359 | MH011379 | MH842103 | MH842104 | MH475084 | MH475085 |
| ROYA63 | Macquarie I. | MH011362 | MH011375 | MH842105 | MH842106 | MH475086 | MH475087 |
| ROYA64 | Macquarie I. | MH011358 | MH011373 | MH842107 | MH842108 | MH475088 | MH475089 |
| ROYA65 | Macquarie I. | MH011363 | MH011377 | MH842109 | MH842110 | MH475090 | MH475091 |
| ROYA66 | Macquarie I. | MH011364 | MH011376 | MH842111 | MH842112 | MH475092 | MH475093 |
| ROYA67 | Macquarie I. | MH011361 | MH011378 | MH842113 | MH842114 | MH475094 | MH475095 |
| ROYA68 | Macquarie I. | MH011360 | MH011374 | MH842115 | MH842116 | MH475096 | MH475097 |
| ROYA69 | Macquarie I. | MH011357 | MH011380 | MH842117 | MH842118 | MH475098 | MH475099 |

**Table S3**. a) Fst values (below diagonal) and corrected p values (above diagonal) for pairwise Fst, and b) ɸst values (below diagonal) and corrected P values (above diagonal) from mtDNA HVR1, for the six locations of macaroni, royal and white-faced penguins. MAR1 denotes white-faced penguins from Marion and MACQ correspond to royal penguins from Macquarie Island. Significant P values (< 0.05) with *.

a)

|  | MACQ | MARI1 | CROZ | MARI | KERG | BOUV | BIRD | ELEP |
| --- | --- | --- | --- | --- | --- | --- | --- | --- |
| MACQ |  | 0.199 | 0.199 | 0.199 | 0.565 | 0.353 | 0.999 | 0.344 |
| MARI1 | 0.03039 |  | 0.402 | 0.199 | 0.199 | 0.199 | 0.216 | 0.199 |
| CROZ | 0.05980 | 0.30155 |  | 0.216 | 0.380 | 0.144 | 0.341 | 0.199 |
| MARI | 0.08554 | 0.04762 | 0.10088 |  | 0.478 | 0.144 | 0.385 | 0.199 |
| KERG | 0.48470 | 0.07128 | 0.25829 | 0.39313 |  | 0.199 | 0.629 | 0.275 |
| BOUV | 0.22750 | 0.06564 | 0.00990 | 0.01030 | 0.03762 |  | 0.436 | 0.999 |
| BIRD | 0.99990 | 0.10801 | 0.19493 | 0.27562 | 0.58489 | 0.34313 |  | 0.591 |
| ELEP | 0.20919 | 0.06277 | 0.02208 | 0.08296 | 0.14761 | 0.99990 | 0.52817 |  |

b)

|  | MACQ | MARI1 | CROZ | MARI | KERG | BOUV | BIRD | ELEP |
| --- | --- | --- | --- | --- | --- | --- | --- | --- |
| MACQ |  | 0.022* | 0.000* | 0.000* | 0.002* | 0.000* | 0.000* | 0.000* |
| MARI1 | 0.26973 |  | 0.053 | 0.176 | 0.6680 | 0.430 | 0.387 | 0.330 |
| CROZ | 0.54501 | 0.24047 |  | 0.247 | 0.014* | 0.001* | 0.055 | 0.009* |
| MARI | 0.45281 | 0.11174 | 0.01355 |  | 0.060 | 0.010* | 0.130 | 0.057 |
| KERG | 0.24127 | -0.03292 | 0.07319 | 0.03891 |  | 0.047* | 0.176 | 0.302 |
| BOUV | 0.32333 | 0.04024 | 0.20995 | 0.15041 | 0.09225 |  | 0.823 | 0.823 |
| BIRD | 0.33696 | -0.01255 | 0.17031 | 0.09822 | 0.05255 | -0.07998 |  | 0.823 |
| ELEP | 0.35409 | 0.02769 | 0.10738 | 0.06810 | 0.01917 | -0.05202 | -0.07105 |  |

**Table S4**. a) Fst values (below diagonal) and corrected p values (above diagonal) for pairwise Fst, and b) ɸst values (below diagonal) and corrected P values (above diagonal) from nuclear AKlong, for the six locations of macaroni, royal and white-faced penguins. MAR1 denotes white-faced penguins from Marion and MACQ correspond to royal penguins from Macquarie Island. Significant P values (< 0.05) with *.

a)

|  | MACQ | MARI1 | CROZ | MARI | KERG | BOUV | BIRD | ELEP |
| --- | --- | --- | --- | --- | --- | --- | --- | --- |
| MACQ |  | 0.006* | 0.098 | 0.098 | 0.165 | 0.022* | 0.005* | 0.098 |
| MARI1 | 0.15143 |  | 0.004* | 0.004* | 0.008 | 0.004* | 0.004* | 0.004* |
| CROZ | 0.02029 | 0.16337 |  | 0.165 | 0.237 | 0.006* | 0.053 | 0.025* |
| MARI | 0.01375 | 0.15213 | 0.00790 |  | 0.186 | 0.191 | 0.112 | 0.133 |
| KERG | 0.01446 | 0.16203 | 0.00651 | 0.00758 |  | 0.006* | 0.006* | 0.006* |
| BOUV | 0.02671 | 0.17241 | 0.03760 | 0.00793 | 0.04847 |  | 0.447 | 0.355 |
| BIRD | 0.04299 | 0.19269 | 0.03067 | 0.01581 | 0.05967 | 0.00100 |  | 0.303 |
| ELEP | 0.02058 | 0.17714 | 0.03160 | 0.01212 | 0.05255 | 0.00422 | 0.00695 |  |

b)

|  | MACQ | MARI1 | CROZ | MARI | KERG | BOUV | BIRD | ELEP |
| --- | --- | --- | --- | --- | --- | --- | --- | --- |
| MACQ |  | 0.019* | 0.33 | 0.296 | 0.150 | 0.335 | 0.530 | 0.324 |
| MARI1 | 0.23292 |  | 0.002* | 0.002* | 0.001* | 0.001* | 0.028* | 0.001* |
| CROZ | 0.00996 | 0.25458 |  | 0.33 | 0.315 | 0.169 | 0.368 | 0.150 |
| MARI | 0.01543 | 0.29048 | 0.00607 |  | 0.150 | 0.33 | 0.459 | 0.33 |
| KERG | 0.03291 | 0.33071 | 0.01185 | 0.02285 |  | 0.150 | 0.099 | 0.057 |
| BOUV | 0.01170 | 0.31700 | 0.02610 | 0.00866 | 0.03172 |  | 0.368 | 0.958 |
| BIRD | -0.00402 | 0.20799 | 0.00628 | 0.00025 | 0.05063 | 0.00770 |  | 0.713 |
| ELEP | 0.01792 | 0.30149 | 0.03146 | 0.01272 | 0.05009 | -0.03252 | -0.01696 |  |

**Table S5.** a) Fst values (below diagonal) and corrected p values (above diagonal) for pairwise Fst, and b) ɸst values (below diagonal) and corrected P values (above diagonal) from mtDNA HVR1 for the 10 locations for rockhopper penguins. Significant P values (< 0.05) with *.

a)

|  | NIGH | AMST | CROZ | MARI | KERG | MACQ | FALK | SANJ | FRAN | TERH |
| --- | --- | --- | --- | --- | --- | --- | --- | --- | --- | --- |
| NIGH |  | 0.003* | 0.008* | 0.074 | 0.006* | 0.328 | 0.003* | 0.002* | 0.003* | 0.010* |
| AMST | 0.03793 |  | 0.000* | 0.013* | 0.000* | 0.115 | 0.002* | 0.001* | 0.000* | 0.011* |
| CROZ | 0.02771 | 0.04556 |  | 0.856 | 0.366 | 0.740 | 0.002* | 0.001* | 0.000* | 0.008* |
| MARI | 0.01350 | 0.03202 | -0.00931 |  | 0.300 | 0.999 | 0.006* | 0.002* | 0.017* | 0.022* |
| KERG | 0.05064 | 0.06796 | 0.00240 | 0.00560 |  | 0.399 | 0.003* | 0.001* | 0.000* | 0.007* |
| MACQ | 0.01074 | 0.03111 | -0.01191 | -0.01304 | 0.00389 |  | 0.087 | 0.074 | 0.168 | 0.243 |
| FALK | 0.06110 | 0.07887 | 0.06866 | 0.05502 | 0.09275 | 0.05707 |  | 0.003* | 0.003* | 0.017* |
| SANJ | 0.09935 | 0.11615 | 0.10611 | 0.09337 | 0.13126 | 0.10160 | 0.14812 |  | 0.074 | 0.074 |
| FRAN | 0.03913 | 0.05705 | 0.04694 | 0.03309 | 0.07009 | 0.03234 | 0.08153 | 0.06039 |  | 0.873 |
| TERH | 0.05932 | 0.07800 | 0.06730 | 0.05295 | 0.09251 | 0.05477 | 0.10657 | 0.10177 | -0.03229 |  |

b)

|  | NIGH | AMST | CROZ | MARI | KERG | MACQ | FALK | SANJ | FRAN | TERH |
| --- | --- | --- | --- | --- | --- | --- | --- | --- | --- | --- |
| NIGH |  | 0.000* | 0.000* | 0.000* | 0.000* | 0.000* | 0.000* | 0.000* | 0.000* | 0.000* |
| AMST | 0.40070 |  | 0.000* | 0.000* | 0.000* | 0.000* | 0.000* | 0.000* | 0.000* | 0.000* |
| CROZ | 0.82194 | 0.81103 |  | 0.0787 | 0.619 | 0.620 | 0.000* | 0.000* | 0.000* | 0.000* |
| MARI | 0.79596 | 0.78550 | 0.06224 |  | 0.000* | 0.193 | 0.000* | 0.000* | 0.000* | 0.000* |
| KERG | 0.84478 | 0.83013 | -0.01048 | 0.11932 |  | 0.799 | 0.000* | 0.000* | 0.000* | 0.000* |
| MACQ | 0.86486 | 0.83624 | -0.02090 | 0.05055 | -0.03904 |  | 0.000* | 0.000* | 0.000* | 0.000* |
| FALK | 0.90736 | 0.88790 | 0.74982 | 0.73566 | 0.77705 | 0.80229 |  | 0.000* | 0.000* | 0.000* |
| SANJ | 0.90020 | 0.87899 | 0.73842 | 0.71470 | 0.77096 | 0.78629 | 0.57269 |  | 0.883 | 0.818 |
| FRAN | 0.89235 | 0.87824 | 0.74811 | 0.72988 | 0.77561 | 0.78909 | 0.56083 | -0.03068 |  | 0.91 |
| TERH | 0.89181 | 0.87126 | 0.71261 | 0.68371 | 0.74628 | 0.75375 | 0.52506 | -0.03868 | -0.04938 |  |

**Table S6**. a) Fst values (below diagonal) and corrected P values (above diagonal) for pairwise Fst, and b) ɸst values (below diagonal) and corrected P values (above diagonal) from nuclear AKlong for the 10 locations of rockhopper penguins. Significant P values (< 0.05) with *.

|  | NIGH | AMST | CROZ | MARI | KERG | MACQ | FALK | SANJ | FRAN | TERH |
| --- | --- | --- | --- | --- | --- | --- | --- | --- | --- | --- |
| NIGH |  | 0.030* | 0.000* | 0.000* | 0.000* | 0.000* | 0.000* | 0.000* | 0.000* | 0.027* |
| AMST | 0.05069 |  | 0.000* | 0.000* | 0.000* | 0.000* | 0.000* | 0.000* | 0.000* | 0.005* |
| CROZ | 0.13064 | 0.19335 |  | 0.355 | 0.710 | 0.139 | 0.002* | 0.003* | 0.001* | 0.027* |
| MARI | 0.16949 | 0.23760 | 0.00417 |  | 0.316 | 0.660 | 0.006* | 0.013* | 0.001* | 0.025* |
| KERG | 0.17339 | 0.22410 | -0.00664 | 0.00624 |  | 0.185 | 0.002* | 0.004* | 0.001* | 0.023* |
| MACQ | 0.22733 | 0.29203 | 0.02726 | -0.02183 | 0.02133 |  | 0.001* | 0.002* | 0.000* | 0.014* |
| FALK | 0.15211 | 0.20928 | 0.08340 | 0.11098 | 0.09438 | 0.17395 |  | 0.031* | 0.255 | 0.255 |
| SANJ | 0.16653 | 0.22431 | 0.09409 | 0.10987 | 0.10464 | 0.18671 | 0.06337 |  | 0.034* | 0.115 |
| FRAN | 0.14146 | 0.19293 | 0.05946 | 0.08408 | 0.06646 | 0.12436 | 0.01306 | 0.04620 |  | 0.255 |
| TERH | 0.19773 | 0.26324 | 0.13691 | 0.19375 | 0.15494 | 0.27158 | 0.04414 | 0.11111 | 0.02989 |  |

a)

b)

|  | NIGH | AMST | CROZ | MARI | KERG | MACQ | FALK | SANJ | FRAN | TERH |
| --- | --- | --- | --- | --- | --- | --- | --- | --- | --- | --- |
| NIGH |  | 0.071 | 0.000* | 0.000* | 0.000* | 0.000* | 0.000* | 0.000* | 0.000 | 0.000* |
| AMST | 0.03667 |  | 0.000* | 0.000* | 0.000* | 0.000* | 0.000* | 0.000* | 0.000 | 0.000* |
| CROZ | 0.53036 | 0.57729 |  | 0.488 | 0.736 | 0.876 | 0.000* | 0.000* | 0.000 | 0.000* |
| MARI | 0.52001 | 0.56516 | -0.00175 |  | 0.103 | 0.824 | 0.000* | 0.001* | 0.000 | 0.000* |
| KERG | 0.58190 | 0.62285 | -0.00793 | 0.03069 |  | 0.578 | 0.000* | 0.000* | 0.000 | 0.000* |
| MACQ | 0.56274 | 0.60854 | -0.02577 | -0.03297 | -0.00740 |  | 0.000* | 0.000* | 0.000 | 0.000* |
| FALK | 0.43797 | 0.47871 | 0.51816 | 0.48347 | 0.55715 | 0.49760 |  | 0.101 | 0.337 | 0.628 |
| SANJ | 0.27469 | 0.29110 | 0.30768 | 0.23445 | 0.35853 | 0.26905 | 0.09146 |  | 0.197 | 0.286 |
| FRAN | 0.32652 | 0.36238 | 0.36651 | 0.31364 | 0.39991 | 0.31713 | 0.00817 | 0.03578 |  | 0.539 |
| TERH | 0.48371 | 0.50395 | 0.61249 | 0.63031 | 0.65452 | 0.66144 | -0.04198 | 0.08745 | -0.03198 |  |

**Table S7**. Neutrality test for macaroni and rockhopper penguins calculated from mtDNA (COI) and nuclear (ODC and AK) markers. MARI^1^ correspond to white-faced penguins from Marion Island. *p < 0.05, ** p < 0.01, *** p < 0.001.

|  |  | COI | | AK | | ODC | |
| --- | --- | --- | --- | --- | --- | --- | --- |
| Species | Location | D | Fs | D | Fs | D | Fs |
| *E. schlegeli* | MACQ | -0.53 | -0.46 | -0.35 | -5.92** | 0.09 | 0.23 |
| *white-faced1* | MARI^1^ | 0.00 | 0.00 | -1.29 | 0.89 | 2.00 | 1.83 |
| *E. chrysolophus* | CROZ | -1.57 | -2.82** | -0.69 | -9.18** | -0.45 | -1.23 |
|  | MARI | -2.05* | -6.91*** | -0.47 | -18.34*** | -0.02 | -0.84 |
|  | KERG | -1.60 | -2.90** | -0.81 | -8.56** | -0.20 | -1.33 |
|  | BOUV | -0.84 | -0.72 | -0.07 | -9.85** | 0.26 | -0.76 |
|  | BIRD | -0.43 | 1.25 | -0.17 | -5.28** | -0.51 | -2.56 |
|  | ELEP | -1.62 | -2.12** | 0.04 | -7.38** | -0.00 | -2.25 |
| *E. moseleyi* | NIGH | 0.00 | 0.00 | -0.16 | -0.88 | -1.29 | -2.02* |
|  | AMST | -1.51 | -2.12* | -0.46 | -1.46 | -1.28 | -1.50 |
| *E. filholi* | CROZ | 0.35 | 1.17 | -0.93 | -4.94 | -0.60 | -1.33 |
|  | MARI | 0.99 | 0.69 | -0.82 | -1.62 | -0.75 | -3.07 |
|  | KERG | 0.07 | 1.28 | -1.12 | -5.57 | 0.11 | -0.92 |
|  | MACQ | 1.58 | 2.53 | -0.75 | 0.52 | -0.61 | -2.05 |
| *E. chrysocome* | FALK | -1.95* | -2.03 | 0.76 | -1.11 | 0.89 | 0.32 |
|  | SANJ | -0.65 | 0.20 | 0.33 | -1.28 | -0.93 | -3.28* |
|  | FRAN | -1.88* | -2.26* | 1.23 | -3.44 | 0.03 | -1.61 |
|  | TERH | -1.31 | 0.76 | 1.36 | 0.81 | -1.54 | -3.47* |

**Table S8**. P values and corrected p values for Tajima’s test (D), Fu’s test (*Fs*) for a) mtDNA HVRI and COI, and b) nuclear AKlong and ODC in macaroni, royal and rockhopper penguins. NC denotes not corrected P values. Significant Pvalues (< 0.05) with *.

| b) |  | |  | **AKlong** |  |  |  | **ODC** |  |  |
| --- | --- | --- | --- | --- | --- | --- | --- | --- | --- | --- |
| Specie | | Location | p value (D) | Corrected p (D) | p value (Fs) | Corrected p (Fs) | p value (D) | Corrected p (D) | p value (Fs) | Corrected p (Fs) |
| *E. schlegeli* | | MACQ | 0.394 | NC | 0.004* | 0.005* | 0.582 | NC | 0.574 | NC |
| *white-faced1* | | MARI1 | 0.077 | NC | 0.661 | 0.661 | 0.992 | NC | 0.841 | NC |
| *E. chrysolophus* | | CROZ | 0.263 | NC | 0.000* | 0.001* | 0.365 | NC | 0.273 | NC |
|  | | MARI | 0.367 | NC | 0.000* | 0.000* | 0.548 | NC | 0.375 | NC |
|  | | KERG | 0.223 | NC | 0.000* | 0.001* | 0.469 | NC | 0.252 | NC |
|  | | BOUV | 0.517 | NC | 0.000* | 0.001* | 0.654 | NC | 0.334 | NC |
|  | | BIRD | 0.471 | NC | 0.005* | 0.005* | 0.340 | NC | 0.068 | NC |
|  | | ELEP | 0.576 | NC | 0.000* | 0.001* | 0.530 | NC | 0.074 | NC |
| *E. moseleyi* | | NIGH | 0.484 | NC | 0.338 | 0.422 | 0.059 | 0.256 | 0.012* | 0.04* |
|  | | AMST | 0.370 | NC | 0.239 | 0.368 | 0.077 | 0.256 | 0.161 | 0.268 |
| *E. filholi* | | CROZ | 0.182 | NC | 0.011 | 0.055 | 0.312 | 0.445 | 0.246 | 0.307 |
|  | | MARI | 0.227 | NC | 0.134 | 0.335 | 0.256 | 0.445 | 0.041* | 0.102 |
|  | | KERG | 0.120 | NC | 0.005* | 0.05 | 0.589 | 0.654 | 0.330 | 0.366 |
|  | | MACQ | 0.245 | NC | 0.628 | 0.628 | 0.293 | 0.445 | 0.099 | 0.198 |
| *E. chrysocome* | | FALK | 0.794 | NC | 0.258 | 0.368 | 0.825 | 0.825 | 0.585 | 0.585 |
|  | | SANJ | 0.664 | NC | 0.216 | 0.368 | 0.193 | 0.445 | 0.012* | 0.04* |
|  | | FRAN | 0.906 | NC | 0.084 | 0.28 | 0.564 | 0.654 | 0.193 | 0.275 |
|  | | TERH | 0.875 | NC | 0.592 | 0.628 | 0.049* | 0.256 | 0.002* | 0.02* |

| a) |  | |  | **HVRI** |  |  |  | **COI** |  |  |
| --- | --- | --- | --- | --- | --- | --- | --- | --- | --- | --- |
| Species | | Location | p value (D) | Corrected p (D) | p value (Fs) | Corrected p (Fs) | p value (D) | Corrected p (D) | p value (Fs) | Corrected p (Fs) |
| *E. schlegeli* | | MACQ | 0.377 | 0.603 | 0.000* | 0.000* | 0.279 | 0.350 | 0.251 | 0.292 |
| *white-faced1* | | MARI1 | 1.000 | 1.000 | 0.807 | 0.807 | 1.000 | 1 | N.A. | NC |
| *E. chrysolophus* | | CROZ | 0.011* | 0.088 | 0.001* | 0.002* | 0.040* | 0.08 | 0.001* | 0.003* |
|  | | MARI | 0.024* | 0.096 | 0.001* | 0.002* | 0.004* | 0.032* | 0.000* | 0* |
|  | | KERG | 0.114 | 0.304 | 0.014* | 0.028* | 0.033* | 0.08 | 0.002* | 0.004* |
|  | | BOUV | 0.592 | 0.676 | 0.255 | 0.316 | 0.238 | 0.350 | 0.086 | 0.12 |
|  | | BIRD | 0.588 | 0.676 | 0.277 | 0.316 | 0.307 | 0.350 | 0.677 | 0.677 |
|  | | ELEP | 0.343 | 0.603 | 0.107 | 0.171 | 0.036 | 0.08 | 0.004* | 0.007* |
| *E. moseleyi* | | NIGH | 0.029* | 0.145 | 0.000* | 0.000* | 1.000 | 1.000 | N.A. | NC |
|  | | AMST | 0.062 | 0.155 | 0.009* | 0.024* | 0.038 | 0.126 | 0.007* | 0.031* |
| *E. filholi* | | CROZ | 0.419 | 0.465 | 0.010* | 0.024* | 0.671 | 0.958 | 0.740 | 0.866 |
|  | | MARI | 0.539 | 0.539 | 0.001* | 0.005* | 0.849 | 1.000 | 0.667 | 0.866 |
|  | | KERG | 0.254 | 0.362 | 0.047* | 0.067 | 0.573 | 0.955 | 0.770 | 0.866 |
|  | | MACQ | 0.300 | 0.375 | 0.044* | 0.067 | 0.953 | 1.000 | 0.903 | 0.903 |
| *E. chrysocome* | | FALK | 0.054 | 0.155 | 0.066 | 0.082 | 0.008* | 0.04* | 0.018* | 0.054 |
|  | | SANJ | 0.120 | 0.24 | 0.601 | 0.601 | 0.286 | 0.572 | 0.442 | 0.866 |
|  | | FRAN | 0.018* | 0.145 | 0.012* | 0.024* | 0.008* | 0.04* | 0.007* | 0.031* |
|  | | TERH | 0.159 | 0.265 | 0.294 | 0.326 | 0.099 | 0.2475 | 0.483 | 0.866 |

**Figure S3**.- Phylogenetic reconstruction of *Eudyptes* species, using nuclear (ODC, AK) and mtDNA (RC, COI) markers (SS = 126, PSRF = 1.006).

**
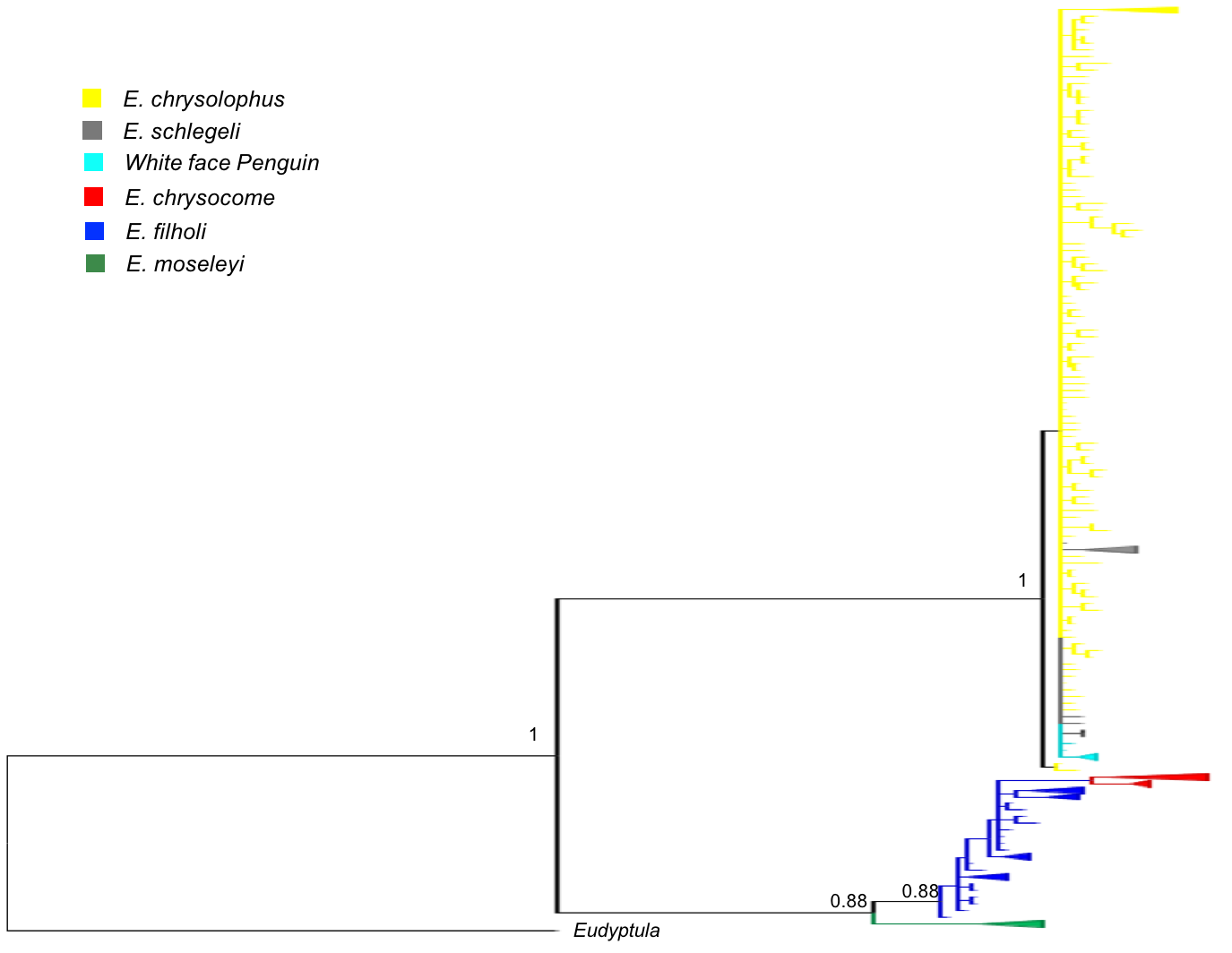
**

**Table S9**. Substitution model for mtDNA HVRI for each location and species of *Eudyptes* penguin.

| Species | Location | Mutation model |
| --- | --- | --- |
| *E. moseleyi* | Amsterdam | HKY+I |
|  | Nightingale | HKY |
| *E. filholi* | Kerguelen | HKY+I |
|  | Crozet | HKY+I |
|  | Marion | HKI+I |
| *E. chrysocome* | Falkland/Malvinas | HKY |
|  | Franklin | HKI |
|  | San Juan | HKI |
|  | Terhalten | HKI+I |
| *E. chrysolophus* | Crozet-Marion | HKY+I |
|  | Kerguelen | HKI+I+G |
|  | Bird-Bouvet-Elephant | HKY+I |
